# Two-dimensional lead-free double perovskite ferroelastics with dynamic thermochromism

**DOI:** 10.1039/d5sc05158d

**Published:** 2025-11-03

**Authors:** Chang-Yuan Su, Heng-Guan Yi, Hao-Fei Ni, Guo-Wei Du, San-Qiang Xia, Zunqi Liu, Zhi-Xu Zhang, Da-Wei Fu

**Affiliations:** a Shanxi Key Laboratory for Radiation Safety and Protection, China Institute for Radiation Protection Taiyuan 030006 China scywmy@163.com; b Institute for Science and Applications of Molecular Ferroelectrics, Key Laboratory of the Ministry of Education for Advanced Catalysis Materials, Zhejiang Normal University Jinhua 321004 China zhangzhixu@zjnu.edu.cn dawei@zjnu.edu.cn; c CNNC Key Laboratory for Radiation Protection Technology, China Institute for Radiation Protection Taiyuan 030006 China; d Jiangsu Key Laboratory of Advanced Laser Materials and Devices, School of Physics and Electronic Engineering, Jiangsu Normal University Xuzhou 221116 China; e Chemistry and Chemical Engineering College, Xinjiang Agricultural University Urumqi 830052 China lzq@xjau.edu.cn

## Abstract

Ferroelastics, as a key branch of ferroic materials, are vital for mechanical switches, energy conversion, *etc.* While research on ferroelastic organic–inorganic hybrid perovskites has laid a preliminary foundation, in-depth exploration is still needed to expand their material system and functional integration. In this work, we constructed two perovskite ferroelastics using cyclobutylmethanaminium (CBA^+^) as the cationic template. First, the lead-based (CBA)_2_PbBr_4_ exhibits a ferroelastic phase transition at Curie temperature (*T*_c_) = 383.4 K (*mmm*F2/*m*). To address lead toxicity, we developed the lead-free double perovskite (CBA)_4_AgBiBr_8_ (*T*_c_ = 346.7 K, 4/*mmm*F2/*m*), which not only achieves lead-free design and increases domain states from 2 to 4, but also features a narrower bandgap (2.22 eV *vs.* 2.97 eV in (CBA)_2_PbBr_4_). Most notably, (CBA)_4_AgBiBr_8_ shows thermochromic behavior, which is rarely observed in hybrid double perovskites. These findings not only expand the perovskite ferroelastic family but also provide a strategy for integrating lead-free design with functional properties like thermochromism.

## Abstract 2

Compared to perovskite (CBA)_2_PbBr_4_, lead-free double perovskite (CBA)_4_AgBiBr_8_ has narrower bandgap, more domains, and thermochromism.

## Graphical abstract

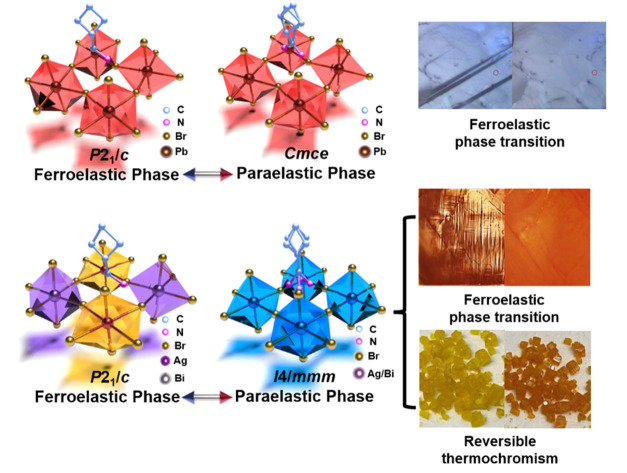

## Introduction

In recent years, thanks to their excellent performance in photovoltaics, ferroelectricity, photodetection, light-emitting diodes and so on, organic–inorganic hybrid perovskites have become one of the hot research topics.^1–8^ In these studies, due to the advantages of high charge-carrier mobility, narrow and tunable bandgaps, high absorption coefficient, *etc.*, lead-based perovskites account for a considerable proportion.^9–13^ However, the toxicity of lead-based perovskites and their long-term instability pose significant limitations to their further development. The potential adverse effects of these materials on animals, plants, and the environment are a major cause for concern and must be addressed.^14,15^

The homo-valent and hetero-valent replacement at inorganic sites can be used to construct lead-free perovskites. The homo-valent replacement refers to the use of metal ions with a +2 valence state (Ge^2+^, Sn^2+^, Cd^2+^, Ba^2+^, *etc.*),^16–20^ which largely maintain good physical properties, but their chemical richness and stability are often questioned. In comparison, the hetero-valent replacement adopts ion-splitting (A_2_B^I^B^III^X_6_) and ordered vacancies (A_3_□B^III^X_9_, A_2_□B^IV^X_6_, *etc.*, □ refers to the vacancy), wherein B^I^-site cations mainly include alkali metals and group IB elements, B^III^-site cations are abundant and can be located in group A and group B, and X at the corner comprises halogens, CN^−^ and NO_3_^−^.^21–27^ It can not only maintain charge neutrality, but also provide a platform for diverse properties, showing better potential waiting for us to explore.^28–33^

Ferroelastics, which are the sister ferroic materials of ferroelectrics and ferromagnetics, can exhibit two or more strain states without mechanical stress, and under the application of mechanical stress, the strain state can transform from one to another.^34^ This material plays an important role in fields such as mechanical switches, shape memory, and the enhancement of key material properties (*e.g.*, piezoelectric properties)—such application prospects have spurred research progress in ferroelastic materials, including organic–inorganic hybrid perovskites.^7,21–24,35–39^ As a prominent example of the hetero-valent replacement, two-dimensional double perovskites (A_4_B^I^B^III^X_8_) have witnessed significant research expansion in various fields in recent years.^40–42^ However, there are few reports on the research of two-dimensional double perovskite ferroelastics, especially in their design and construction and multifunctional integration. Considering the demand for such multifunctional integration, thermochromism—as another functional property that could enrich the multifunctional potential of perovskites—is worth attention: in organic–inorganic hybrid perovskites, thermochromic behavior has been reported in multiple systems such as lead-based and copper-based ones (*e.g.*, MHy_2_PbBr_4_,^12^ MHyPbBr_3_,^43^ PEA_2_PbBr_4_,^44^ [3,3-difluorocyclobutylammonium]_2_CuCl_4_,^45^ (BEA)_2_CuCl_4_,^46^*etc.*), yet for double perovskites, this property is only found in inorganic systems (*i.e.*, Cs_2_AgBiBr_6_ and Cs_2_NaFeCl_6_) and a single hybrid double perovskite case ((H_2_MPP)_2_[BiAgI_8_]).^47–49^ This obvious research gap further motivates us to explore thermochromism-integrated 2D double perovskite ferroelastics.

**Scheme 1:**
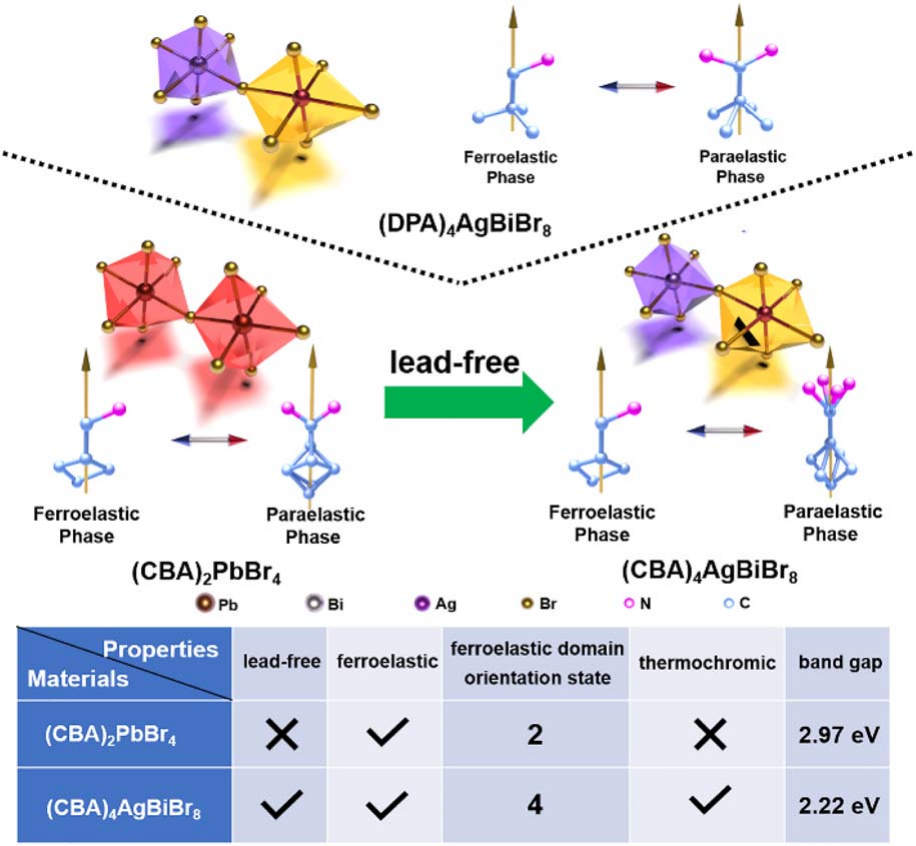
Taking inspiration from (DPA)_4_AgBiBr_8_, two new perovskite ferroelastics ((CBA)_2_PbBr_4_ and (CBA)_4_AgBiBr_8_) were successfully constructed. More importantly, the lead-free (CBA)_4_AgBiBr_8_ not only has more ferroelastic domain states (4 *vs.* 2) and a narrower bandgap (2.22 eV *vs.* 2.97 eV) than (CBA)_2_PbBr_4_, but also exhibits thermochromic behavior.

In 2022, our team successfully constructed a hybrid double perovskite ferroelastic (DPA)_4_AgBiBr_8_ (DPA^+^ = 2,2-dimethylpropan-1-aminium) with high Curie temperature (*T*_c_), using DPA^+^ as a cation and (AgBr_6_)^5−^ and (BiBr_6_)^3−^ as two-dimensional inorganic frameworks.^50^ In this work, taking inspiration from (DPA)_4_AgBiBr_8_, two new two-dimensional perovskite ferroelastics (CBA)_2_PbBr_4_ (*T*_c_ = 383.4 K, *mmm*F2/*m*) and (CBA)_4_AgBiBr_8_ (*T*_c_ = 346.7 K, 4/*mmm*F2/*m*) were successfully constructed by selecting CBA^+^ (CBA^+^ = cyclobutylmethanaminium) that may rotate and combining it with (PbBr_6_)^4−^ and (AgBr_6_)^5−^ & (BiBr_6_)^3−^ (Scheme 1). It is worth highlighting that the lead-free double perovskite ferroelastic (CBA)_4_AgBiBr_8_ not only increases the domain states from 2 to 4 but also exhibits a reduced band gap from 2.97 eV to 2.22 eV. Most notably, (CBA)_4_AgBiBr_8_ exhibits thermochromic behavior, which is rarely observed in hybrid double perovskites. The discovery of these two double perovskite ferroelastics expands the ferroelastic family. Meanwhile, it offers a viable approach for screening appropriate organic cations and inorganic components, which can spur the exploration of more perovskite ferroelastic materials with functional properties like thermochromism.

## Results and discussion

### Analysis of basic crystal structure and phase transition behaviors

**Fig. 1:**
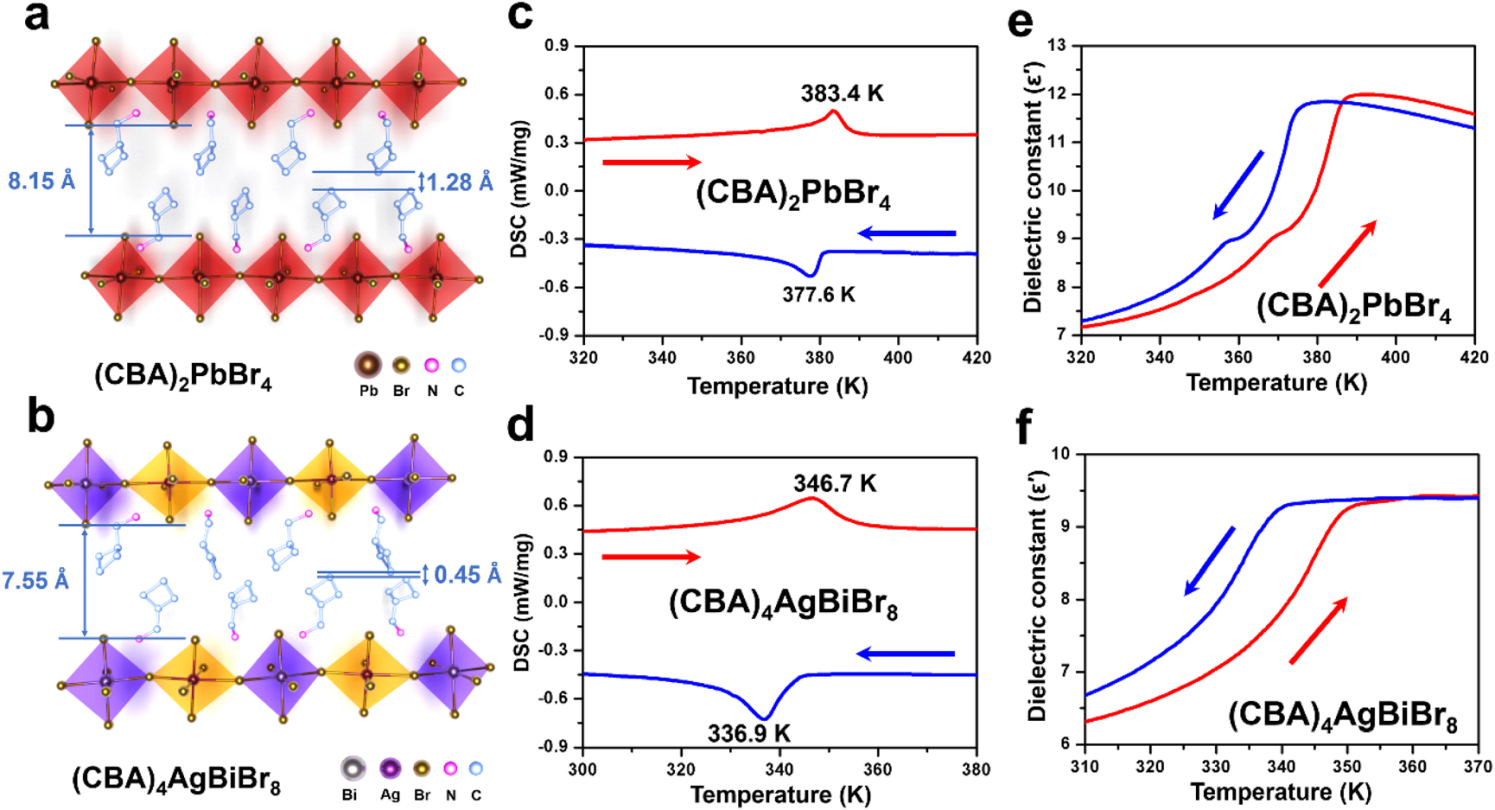
The two-dimensional stacking structures (a and b), DSC profile (c and d), and dielectric constant at 1 MHz (e and f) of (CBA)_2_PbBr_4_ and (CBA)_4_AgBiBr_8_.

Using single crystal X-ray diffraction, the crystal structures of (CBA)_2_PbBr_4_, and (CBA)_4_AgBiBr_8_ were obtained. The crystal data, structure refinements and selected bond information of (CBA)_2_PbBr_4_ and (CBA)_4_AgBiBr_8_ and their crystal data and structure refinements are listed in Tables S1–S4. For (CBA)_2_PbBr_4_, it is located in *P*2_1_/*c* (No. 14) and adopts a 〈100〉-oriented layered corner sharing Ruddlesden–Popper perovskite structure with *n* = 1, in which the N elements in the upper and lower layers respectively face the upper and lower inorganic structures (Fig. 1a). While keeping the CBA^+^ cation unchanged, with the replacement of inorganic basic unit (PbBr_6_)^4−^ with (AgBr_6_)^5−^ & (BiBr_6_)^3−^, the space group of (CBA)_4_AgBiBr_8_ remains unchanged and remains *P*2_1_/*c* (No. 14) (Fig. 1b). Even if both adopt the same space group and stacking method, some structural differences can be easily perceived, such as the interlayer spacing of inorganic frameworks, the interlayer spacing of organic molecules in the upper and lower layers and so on. In addition, the stacking structure containing hydrogen bonds is shown in Fig. S1.

The inorganic frameworks of the two-dimensional hybrid perovskite exhibit the characteristics of an ordered structure and can provide enough space for charge balanced organic cations to realize the possible thermal motion of molecules, which provides the possibility of inducing the order–disorder structural phase transition and leading to the transformation of physical properties. For this, differential scanning calorimetry (DSC) (Fig. 1c and d) and temperature-dependent dielectric measurements (Fig. 1e and f) of (CBA)_2_PbBr_4_ and (CBA)_4_AgBiBr_8_ were conducted. As shown, (CBA)_2_PbBr_4_ and (CBA)_4_AgBiBr_8_ exhibit phase transition behavior including reversible thermal and dielectric anomalies at 383.4 K and 346.7 K, respectively. We further calculated *N* (the ratio of the number of equivalent orientations in the high-temperature phase) for both *via* the Boltzmann equation Δ*S* = *R* ln *N* (*R* is a gas constant): *N* = 5.1 for (CBA)_2_PbBr_4_, which indicates that CBA^+^ cations are in a disordered state and the (PbBr_6_)^4−^ exhibits distortion in the high-temperature phase; in sharp contrast, *N* = 51.8 for (CBA)_4_AgBiBr_8_—this much higher value than that of (CBA)_2_PbBr_4_ suggests that its organic cations tend to be in a more disordered state and the (AgBr_6_)^5−^/(BiBr_6_)^3−^ inorganic framework undergoes more significant deformation.

### Analysis of the variable-temperature crystal structure

For the newly constructed hybrid perovskites (CBA)_2_PbBr_4_ and (CBA)_4_AgBiBr_8_ with a solid-to-solid phase transition, it is necessary to determine their structure in the high-temperature phase to better understand the relationship between the structure and physical properties. At 388 K, (CBA)_2_PbBr_4_ is located in *Cmce* (No. 64) of the orthorhombic crystal system (Table S1). Before the phase transition, the organic part exhibits an ordered molecular configuration, and the inorganic skeleton exhibits distortion and deformation distinct from pure inorganic classes such as CsPbBr_3_, with fewer symmetric elements (Fig. S2a–c). However, in the high-temperature phase (388 K), the organic cations are located at symmetric sites including 2-fold rotoinversion axes and glide planes (Fig. S2d) and present a disordered dual orientation under thermal stimulation. For the inorganic skeleton in the high-temperature phase, there is no significant twisting change overall, and there are slight changes in the bond angle and bond length (Fig. S2e, f and Table S5).

**Fig. 2:**
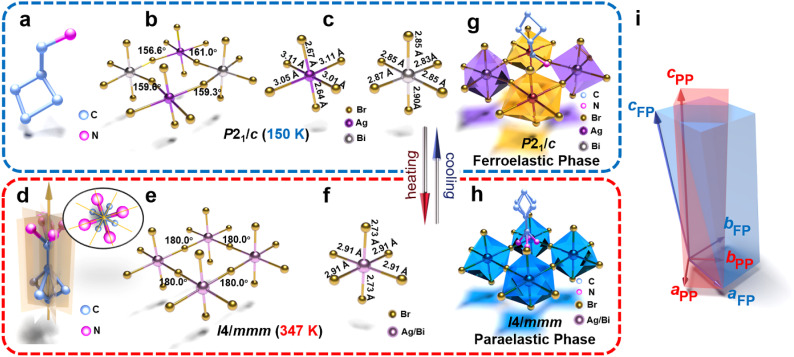
The organic and inorganic components of (CBA)_4_AgBiBr_8_ in ferroelastic (a–c) and paraelastic (d–f) phases. The basic stacking structure of (CBA)_4_AgBiBr_8_ in the ferroelastic (g) and paraelastic (h) phases. (i) Schematic diagram of the relationship between the crystal cell of (CBA)_4_AgBiBr_8_ in the ferroelastic and paraelastic phases. For the convenience of expression, the ferroelastic phase is abbreviated as FP and paraelastic phase is abbreviated as PP.

Unlike the space group *Cmce* (No. 64) of (CBA)_2_PbBr_4_ in the high-temperature phase, (CBA)_4_AgBiBr_8_ crystallizes on *I*4/*mmm* (No. 139) of the tetragonal crystal system with cell parameters *a* = *b* = 5.818(2) Å, *c* = 28.386(15) Å and volume = 960.8 (8) Å^3^ at 347 K (Table S2). Before the phase transition, the organic part exhibits an ordered molecular configuration and the inorganic skeleton exhibits distortion and deformation, possessing fewer symmetric elements (Fig. 2a–c). After the phase transition, the CBA^+^ cations are located at symmetric sites featuring 4-fold rotoinversion axes and multiple groups of mirror and glide planes (Fig. 2d). For inorganic frameworks, the Ag^+^ and Bi^3+^ in the high temperature-phase cannot be distinguished, manifested in a co-occupied form of Ag^+^ & Bi^3+^, with octahedra exhibiting highly symmetric shapes (Fig. 2e, f and Table S6), a feature similar to that in inorganic double perovskites Cs_2_AgBiBr_6_ and Cs_2_AgBiCl_6_.

According to the space group information in the high-temperature and low-temperature phases, (CBA)_2_PbBr_4_ and (CBA)_4_AgBiBr_8_ can be classified as ferroelastic phase transitions with the Aizu notations of *mmm*F2/*m* and 4/*mmm*F2/*m*, respectively. The basic stacking structures of (CBA)_2_PbBr_4_ and (CBA)_4_AgBiBr_8_ in the ferroelastic and paraelastic phases are depicted in Fig. S2g, h, and 2g and h, respectively. In addition, the cell relationships of (CBA)_2_PbBr_4_ and (CBA)_4_AgBiBr_8_ in the ferroelastic and paraelastic phases are shown in Fig. S2i and 2i.

As one of the triggers of the ferroelastic phase transition of these two, CBA^+^ cations exhibiting disordered multi-directional states may undergo rotational motion in the paraelastic phase similar to DPA^+^ cations in (DPA)_4_AgBiBr_8_. Regarding this, the organic cations in the asymmetric units of (DPA)_4_AgBiBr_8_, (CBA)_2_PbBr_4_ and (CBA)_4_AgBiBr_8_ were appropriately rotated and modeled (Fig. S3–S5), and the corresponding rotational energy was calculated (Fig. S6). The results show that the maximum energy required for the rotation of CBA^+^ cations in (CBA)_2_PbBr_4_ is 6.99 eV, which is greater than that of DPA^+^ (4.92 eV) in (DPA)_4_AgBiBr_8_ and CBA^+^ (3.78 eV) in (CBA)_4_AgBiBr_8_. This calculation is consistent with the phase transition temperature shown by DSC and temperature-dependent dielectric measurements, which indirectly reflects the possibility of cation rotation and the rationality of modeling and calculation.

### Ferroelasticity

**Fig. 3:**
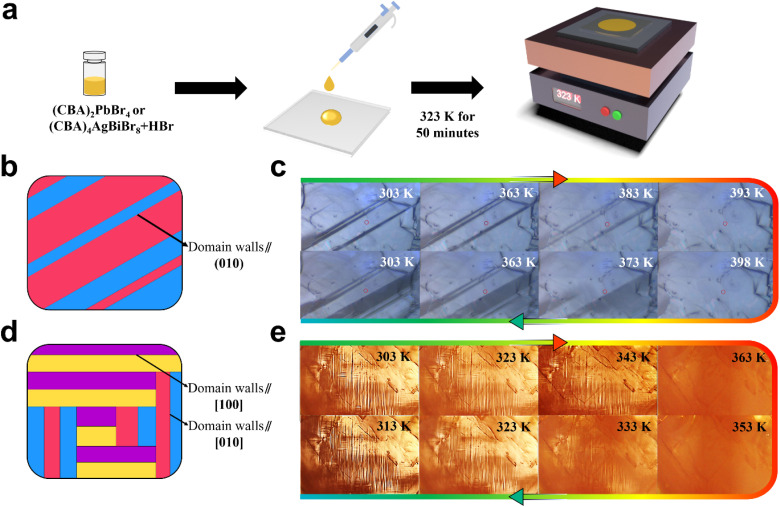
(a) Preparation process of the thin films of (CBA)_2_PbBr_4_ and (CBA)_4_AgBiBr_8_. The schematic diagram and evolution of ferroelastic domains as a function of temperature of (CBA)_2_PbBr_4_ (b and c) and (CBA)_4_AgBiBr_8_ (d and e).

According to the Aizu notations of *mmm*F2/*m* and 4/*mmm*F2/*m*, (CBA)_2_PbBr_4_ and (CBA)_4_AgBiBr_8_ are both typical ferroelastics, whose ferroelastic domains can be observed by using a variable-temperature polarizing microscope. Compared to inorganic materials, as one of the characteristics of organic–inorganic hybrid perovskites, they can be prepared into thin films through a simple solvent evaporation method (Fig. 3a).

The eight symmetric elements of (CBA)_2_PbBr_4_ in the paraelastic phase are E, C_2_, 
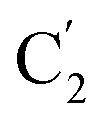
, 
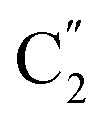
, i, σ_h_, σ_v_ and 
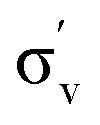
, and the four symmetric elements in the ferroelastic phase are E, C_2_, i and σ_h_. The possible orientation state is *q* = 8/4, *i.e.*, 2. The factor 2 arises because the lost operations (
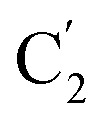
, 
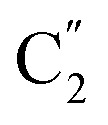
, σ_v_, and 
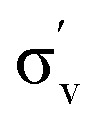
) form conjugate pairs, breaking the *a*–*c* shear-direction freedom. This creates two domains with opposed strains, separated by domain walls in the (010) plane—which contains both *a*- and *c*-axes (Fig. 3b). By observing the thin film of (CBA)_2_PbBr_4_ in the ferroelastic phase, the striped domain walls can be clearly seen. As the temperature gradually increases and exceeds *T*_c_ = 383.4 K, the ferroelastic domains will suddenly disappear, which can be understood as the disappearance of spontaneous strain in the paraelastic phase. But as the temperature decreases, the ferroelastic domains of (CBA)_2_PbBr_4_ will gradually appear again (Fig. 3c). Unlike (CBA)_2_PbBr_4_, the point group of (CBA)_4_AgBiBr_8_ in the paraelastic phase has sixteen symmetric elements: E, 2C_4_, C_2_, 
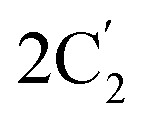
, 
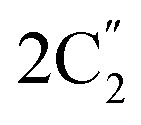
, i, 2S_4_, σ_h_, 2σ_v_ and 2σ_d_, and the ferroelastic phase includes four symmetric elements containing E, C_2_, i and σ_h_. The possible orientation state is *q* = 16/4, that is, 4. This symmetry breaking generates 4 possible orientation states separated by two domain walls: regions with purely horizontal walls (∥*b*-axis, σ_v_-bound) and regions with purely vertical walls (∥*a*-axis, σ_d_-bound), meeting only at T-junctions. Horizontal domain walls result from broken σ_v_ mirrors that were perpendicular to the *a*-axis, whereas vertical walls stem from broken σ_d_ mirrors that were aligned along [110] and [11̄0] directions in the tetragonal system (Fig. 3d). Therefore, in the ferroelastic phase, we observe T-junctioned orthogonal domain walls featuring exclusively T-type intersections without any cross-shaped overlaps as visually distinct horizontal and vertical stripes. Similarly, as the temperature exceeds the Curie temperature of 346.7 K, ferroelastic domains will suddenly disappear and then reappear as the temperature decreases (Fig. 3e).

Notably, the domains separated by domain walls and the “disappearance of strain-related domain contrast” at *T*_c_ are direct consequences of spontaneous strain—a core physical quantity for ferroelastics explicitly defined by Aizu.^34,51^ As Aizu elaborated, it refers to the ferroelastic phase's intrinsic lattice distortion relative to its high-symmetry paraelastic “prototype” phase, distinct from temperature-driven reversible thermal expansion. Spontaneous strain stems from symmetry breaking at *T*_c_ (vanishing above *T*_c_ when the prototype's symmetry is restored) and is described by a symmetric second-rank tensor (*ε*_*ij*_) inheriting both phases' point-group symmetries.^51,52^ This tensor links microscopic distortion to macroscopic domains—for example, (CBA)_2_PbBr_4_'s striped domains come from spontaneous strain tensor differences between its two orientation states, which create domain boundary-forming strain contrast.

The spontaneous strain tensor can be calculated using the following matrix^51,53,54^ (1) according to their Aizu notations of *mmm*F2/*m* and 4/*mmm*F2/*m* from a high-symmetry phase to a low-symmetry phase:1
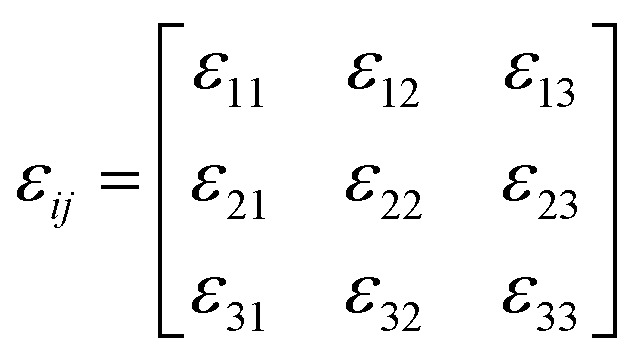
2
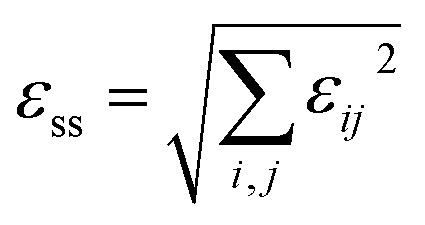


For (CBA)_2_PbBr_4_ (*mmm*F2/*m*), its paraelastic prototype (388 K, *Cmce*) has *a*_PP_ = 29.24 Å, *b*_PP_ = 8.247 Å, *c*_PP_ = 8.249 Å, *α*_PP_ = *β*_PP_ = *γ*_PP_ = 90°, and *Z* = 2, matching the ferroelastic phase's *Z* = 2. We normalize *a*_PP_ to *a*_PP,norm_ = *a*_PP_/2 = 14.62 Å (aligning with the ferroelastic phase's *a*_FP_).^55^ Key strain components include tensile strains (*ε*_11_ ≈ −0.0192, *ε*_22_ ≈ 0.0051, and *ε*_33_ ≈ 0.0021) and shear strain (*ε*_12_ = *ε*_21_ ≈ −0.0853), with other components zero (allowed by 2/*m* symmetry) in the formulae (1).

For (CBA)_4_AgBiBr_8_ (4/*mmm*F2/*m*), its paraelastic prototype (347 K, *I*4/*mmm*) has *a*_PP_ = *b*_PP_ = 5.818 Å, *c*_PP_ = 28.386 Å, *α*_PP_ = *β*_PP_ = *γ*_PP_ = 90°, and *Z* = 1. We scale *a*_PP_ to 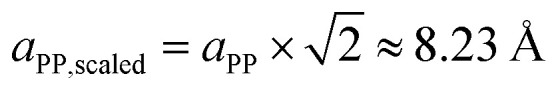 (matching the ferroelastic phase's *Z* = 2). Strain components include tensile strains (*ε*_11_ ≈ −0.0048, *ε*_22_ ≈ 0.005, and *ε*_33_ ≈ −0.0616) and shear strain (*ε*_13_ = *ε*_31_ ≈ −0.0698), with other components zero (forbidden by 4/*mmm*F2/*m* symmetry breaking) in the formulae (1). Eventually, by substituting the parameters in the ferroelastic and paraelastic phases in formulae (1) and (2) (see the SI for details), the total spontaneous strain *ε*_*ss*_ of (CBA)_2_PbBr_4_ and (CBA)_4_AgBiBr_8_ is estimated to be 0.124 and 0.131, respectively. For comparison, reported *ε*_ss_ values of other two-dimensional organic–inorganic hybrid perovskite ferroelastics fall in a similar range: 0.191 ([C_4_H_9_N]_2_[PbBr_4_]), 0.16 ((3-FC_6_H_5_CH_2_CH_2_NH_3_)_2_[CdCl_4_]), 0.156 ((DPA)_4_AgBiBr_8_) and 0.134 ([C_7_H_16_N]_2_[SnI_4_]).^50,56–58^

In addition, the evolution of ferroelastic domain structures of (CBA)_2_PbBr_4_ and (CBA)_4_AgBiBr_8_ was recorded under two conditions: first, during heating/cooling cycles. The temperatures at which the domains disappear and recover are completely consistent with the results from DSC, dielectric, and single crystal X-ray diffraction measurements, showing a clear transformation (Videos S1 and S2, SI). Second, under the application of mechanical stress. Application of stress to the green-ellipsed region leads to a distinct change in ferroelastic domains within the red-ellipsed region, indicating a corresponding change in the strain state (Fig. S7 and S8, SI; note that unlike organic materials, these organic–inorganic hybrid perovskites exhibit brittleness similar to inorganic materials, which may cause slight crystal damage in local areas during mechanical stress application, as observed in the green-ellipsed region).

### Analysis of thermochromism behavior

**Fig. 4:**
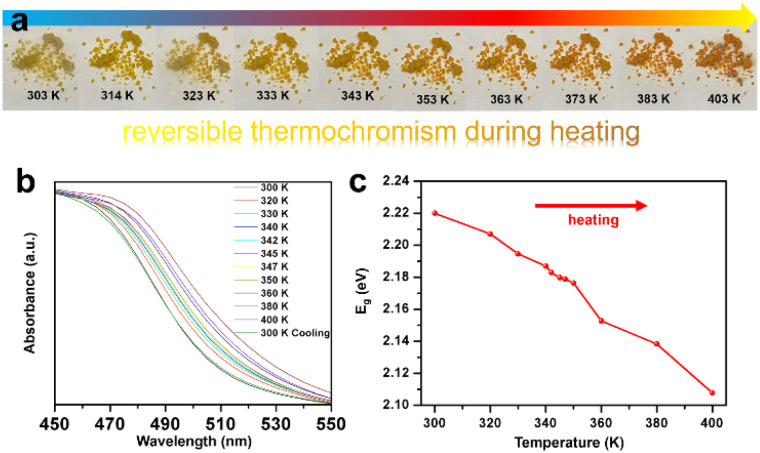
(a) The thermochromic behavior of (CBA)_4_AgBiBr_8_ during heating. The temperature-dependent UV-vis absorbance spectra (b) and corresponding bandgap (c) of (CBA)_4_AgBiBr_8_.

It is worth mentioning that when conducting temperature-dependent tests on the hybrid double perovskite ferroelastic (CBA)_4_AgBiBr_8_, it is noticed that its color changes with temperature, *i.e.*, thermochromism. When heated from room temperature, the material gradually darkens from light yellow at 303 K to orange-red at 403 K (Fig. 4a). Upon cooling back to room temperature, the material fully recovers its initial light yellow state (Fig. S9), demonstrating excellent thermochromic reversibility.

In order to explore the thermochromism behavior of (CBA)_4_AgBiBr_8_, the temperature-dependent UV-vis absorbance spectra were collected over the range of 300–400 K (Fig. 4b and S10). When the temperature increases, the absorption band shows a remarkable red shift from 300 to 400 K: the absorption cut-off edge gradually shifts from 519 nm at 300 K to 537 nm at 400 K. When the temperature is cooled back to 300 K, the absorption cut-off edge recovers to 519 nm, confirming that this hybrid double perovskite exhibits excellent thermochromic reversibility, which is consistent with the observations in Fig. S9. Calculation of the corresponding bandgap values shows that the bandgap narrows gradually with increasing temperature, from 2.22 eV at 300 K to 2.11 eV at 400 K, representing a total bandgap narrowing of 112 meV (Fig. 4c). This redshift is attributed to the reduced tilting and distortion of the inorganic octahedra with increasing temperature. Notably, the bandgap changes more rapidly near the phase transition temperature, with a ∼30 meV decrease occurring in this range that is greater than the ∼20 meV of MHy_2_PbBr_4_ and smaller than the ∼55 meV of MHyPbBr_3_,^12,43^ which can be attributed to the symmetrization of the structure near *T*_c_.^43,45^

Furthermore, the Raman spectroscopy measurements of the (CBA)_4_AgBiBr_8_ were conducted using a 532 nm laser as the excitation source and covering a variable temperature range from 293 K to 413 K (Fig. S11a and b). At 293 K, within the 100–200 cm^−1^ wavenumber range, two prominent peaks are observed at 141.6 cm^−1^ and 162.7 cm^−1^, both assigned to the stretching vibrations of the inorganic octahedra in the perovskite structure.^59–63^ As the temperature increases to 413 K, these two main peaks exhibit an abnormal blueshift, shifting to 143 cm^−1^ and 168.8 cm^−1^ respectively, which is contrary to the expected redshift that results from thermal expansion weakening chemical bonds (Fig. S11c–f). This phenomenon is mainly due to high-temperature-induced disordering of organic CBA^+^ cations, which weakens their directional hydrogen bonds with Br^−^ in the inorganic octahedral layer, reducing the degree of octahedral tilting and distortion and thus elevating the phonon frequency.^59,63^ Meanwhile, this structural regularity is linked to the phase transition-induced statistical co-occupation of Ag^+^ and Bi^3+^: as Ag has a much smaller atomic mass than Bi, the substitution of lighter Ag^+^ in co-occupied octahedra further boosts the phonon vibrational frequency, causing the blueshift.

### Analysis of semiconducting behavior

**Fig. 5:**
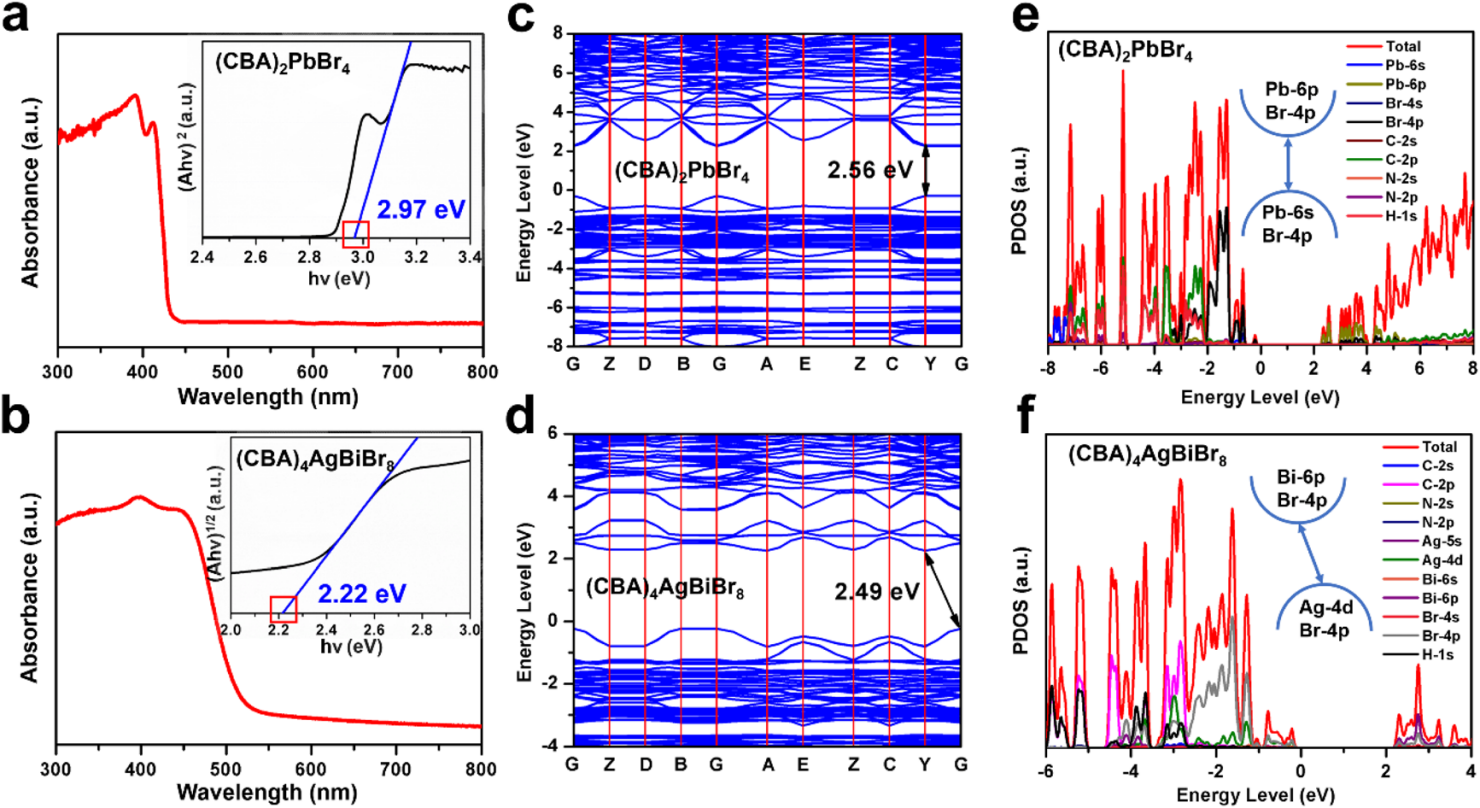
The UV-vis absorbance spectra and corresponding Tauc plots of (CBA)_2_PbBr_4_ (a) and (CBA)_4_AgBiBr_8_ (b). Calculated energy band structures and corresponding partial density of states of (CBA)_2_PbBr_4_ (c and e) and (CBA)_4_AgBiBr_8_ (d and f).

By means of UV-vis absorption and density functional theory (DFT) calculations, the changes in bandgaps of (CBA)_2_PbBr_4_ and (CBA)_4_AgBiBr_8_ are considered in Fig. 5a and b. (CBA)_2_PbBr_4_ and (CBA)_4_AgBiBr_8_ exhibit bandgap values of 2.97 eV and 2.22 eV, respectively. When the number of inorganic perovskite layers (*n*) is 1, for Ruddlesden–Popper-type Pb–Br and Ag&Bi–Br perovskites, the joint action of organic cations and inorganic components leads to distinct structural characteristics—specifically, differences in interlayer distances, degrees of out-of-plane/in-plane octahedral tilting, and other related structural features. These structural disparities further induce variations in the electronic structure of the materials, which in turn result in differences in their bandgaps—*e.g.*, (F_2_CHCH_2_NH_3_)_2_PbBr_4_ (3.2 eV),^64^ MHy_2_PbBr_4_ (3.02 eV),^12^ CHA_2_PbBr_4_ (3.05 eV),^65^ [4,4-DFPD]_2_PbBr_4_ (2.95)^66^ and so on for Pb–Br perovskites (Table S7) and (BA)_4_AgBiBr_8_ (2.5 eV),^67^ (PA)_4_AgBiBr_8_ (2.41 eV),^68^ (OcA)_4_AgBiBr_8_ (2.45 eV)^68^ and (DPA)_4_AgBiBr_8_ (2.44 eV)^50^ and so on for Ag&Bi–Br double perovskites (Table S7).

Using the VASP, the valence band maximum (VBM), the conduction band minimum (CBM) and corresponding partial density of states (PDOS) of (CBA)_2_PbBr_4_ and (CBA)_4_AgBiBr_8_ were calculated to discuss the electronic structure of Pb-based and Ag&Bi-based perovskites (Fig. 5c–f, S12 and S13). Among them, (CBA)_2_PbBr_4_ exhibits a direct bandgap, similar to previously reported Pb-based perovskites, while (CBA)_4_AgBiBr_8_ exhibits an indirect bandgap. As shown in Fig. 5e, the bandgap of (CBA)_2_PbBr_4_ is mainly contributed by inorganic components, with the valence band maximum (VBM) dominated by Pb 6s and Br 4p orbitals and the conduction band minimum (CBM) dominated by Pb 6p orbitals—consistent with the electronic structure characteristics of Pb-based perovskites summarized earlier. Similarly, the bandgap of (CBA)_4_AgBiBr_8_ is mainly derived from its inorganic part: the VBM is primarily composed of Ag 4d and Br 4p orbitals and the CBM is dominated by Bi 6p and Br 4p orbitals (Fig. 5f). In addition, in the newly constructed (CBA)_2_PbBr_4_ and (CBA)_4_AgBiBr_8_, there is a large overlap between the H 1s, N 2p, and C 2p states of organic CBA^+^ cations, indicating a strong interaction within the organic component (especially covalent interactions among H 1s, N 2p, and C 2p states). These organic states do not directly participate in the composition of the VBM and CBM, but their interactions with inorganic frameworks can affect the differences in band gaps.

## Conclusions

In this work, two Ruddlesden–Popper perovskite ferroelastics (CBA)_2_PbBr_4_ (*T*_c_ = 383.4 K, *mmm*F2/*m*) and (CBA)_4_AgBiBr_8_ (*T*_c_ = 346.7 K, 4/*mmm*F2/*m*) were successfully constructed using CBA^+^ as a cation template with (PbBr_6_)^4−^ and (AgBr_6_)^5−^ & (BiBr_6_)^3−^. Their phase transitions stem from ordered-multi-directional disordered CBA^+^ cation transformations and inorganic skeleton distortions. It is worth emphasizing that the lead-free double perovskite ferroelastic (CBA)_4_AgBiBr_8_ not only raises the domain states from 2 to 4, but also shows a reduced band gap (from 2.97 eV to 2.22 eV). Most notably, it exhibits thermochromic behavior. This work enriches perovskite ferroelastic families and offers a strategy for screening cations/inorganic components to explore more such materials.

## Experimental

### Synthesis of single crystals

#### (Cyclobutylmethanaminium)_2_PbBr_4_ (CBA)_2_PbBr_4_

Stoichiometric amounts of cyclobutylmethylamine (0.2 mmol) and lead bromide (0.2 mmol) were added into a beaker. After this, hydrobromic acid (AR ≥ 40%, 20 mL) was added into the beaker. Colorless prism crystals of (CBA)_2_PbBr_4_ were obtained for single crystal X-ray diffraction through slow evaporation of mixed solution at room temperature after several days.

#### (Cyclobutylmethanaminium)_4_AgBiBr_8_ (CBA)_4_AgBiBr_8_

A round-bottom flask containing a turbid liquid of Ag_2_O (0.5 mmol), Bi_2_O_3_ (0.5 mmol), cyclobutylmethanamine (10 mmol) and HBr acid (AR ≥ 40%, 10 mL) was heated at 373 K to obtain a transparent yellow solution. After slowly cooling to room temperature, yellow bulk crystals of (CBA)_4_AgBiBr_8_ were obtained for single crystal X-ray diffraction. Powder X-ray diffraction (PXRD) patterns for verifying the purity of (CBA)_2_PbBr_4_ and (CBA)_4_AgBiBr_8_ are shown in Fig. S14a and b.

### Preparation of thin films

In order to observe the ferroelastic domain structure more clearly, 20 μL of solution containing HBr acid (AR ≥ 40%, 500 μL) and (CBA)_4_AgBiBr_8_ (25 mg) was dripped onto the ozone treated (20 minutes) ITO glass, which was subjected to a heat treatment of 323 K for 50 minutes to obtain thin films subsequently (Fig. 3a). Similarly, the preparation of (CBA)_2_PbBr_4_ thin films only requires replacing (CBA)_4_AgBiBr_8_ (25 mg) with (CBA)_2_PbBr_4_ (25 mg) in the above steps.

## Author contributions

C.-Y. Su conceived and conducted the experiments, analyzed the data and wrote the paper. H.-G. Yi carried out the observation of ferroelastic domains. H.-F. Ni carried out the dielectric characterization studies. G.-W. Du performed temperature-dependent Raman spectroscopy. S.-Q. Xia assisted in data analysis. Z. Liu, Z.-X. Zhang and D.-W. Fu guided and supervised this work.

## Conflicts of interest

The authors declare no conflict of interest.

## Supplementary Material

SC-OLF-D5SC05158D-s001

SC-OLF-D5SC05158D-s002

SC-OLF-D5SC05158D-s003

SC-OLF-D5SC05158D-s004

## Data Availability

CCDC 2286670 ((CBA)_2_PbBr_4_ at 283 K), 2286671 ((CBA)_2_PbBr_4_ at 388 K), 2270990 ((CBA)_4_AgBiBr_8_ at 150 K) and 2270991 ((CBA)_4_AgBiBr_8_ at 347 K) contain the supplementary crystallographic data for this paper.^69*a*–*d*^ The data supporting this article have been included as part of the supplementary information (SI). Supplementary information: Fig. S1–S14, Tables S1–S7, and Videos S1 and S2. See DOI: https://doi.org/10.1039/d5sc05158d.

## References

[cit1] Zou Y., Yu W., Guo H., Li Q., Li X., Li L., Liu Y., Wang H., Tang Z., Yang S., Chen Y., Qu B., Gao Y., Chen Z., Wang S., Zhang D., Chen Y., Chen Q., Zakeeruddin S. M., Peng Y., Zhou H., Gong Q., Wei M., Graetzel M., Xiao L. (2024). Science.

[cit2] Liu C., Yang Y., Chen H., Spanopoulos I., Bati A. S. R., Gilley I. W., Chen J., Maxwell A., Vishal B., Reynolds R. P., Wiggins T. E., Wang Z., Huang C., Fletcher J., Liu Y., Chen L. X., De Wolf S., Chen B., Zheng D., Marks T. J., Facchetti A., Sargent E. H., Kanatzidis M. G. (2024). Nature.

[cit3] Thouin F., Valverde-Chavez D. A., Quarti C., Cortecchia D., Bargigia I., Beljonne D., Petrozza A., Silva C., Kandada A. R. S. (2019). Nat. Mater..

[cit4] Zhang D., Cheng L., Song X., Zhang X., Zhang L., Zhu S., Hu M., Liu Y., Ouyang X., Zheng W. (2025). Angew. Chem., Int. Ed..

[cit5] Xu W., Hu Q., Bai S., Bao C., Miao Y., Yuan Z., Borzda T., Barker A. J., Tyukalova E., Hu Z., Kawecki M., Wang H., Yan Z., Liu X., Shi X., Uvdal K., Fahlman M., Zhang W., Duchamp M., Liu J.-M., Petrozza A., Wang J., Liu L.-M., Huang W., Gao F. (2019). Nat. Photonics.

[cit6] Liu J.-Y., Lun M.-M., Wang Z.-J., Li J.-Y., Ding K., Fu D.-W., Lu H.-F., Zhang Y. (2024). Chem. Sci..

[cit7] Jia Q.-Q., Teri G., Luo J.-Q., Ni H.-F., Huang P.-Z., Lun M.-M., Zhang Z.-X., Zhang Y., Fu D.-W. (2024). J. Am. Chem. Soc..

[cit8] Mączka M., Zaręba J. K., Gągor A., Fedoruk-Piskorska K., Stefańska D., Drozdowski D., Ptak M., Sieradzki A. (2024). ACS Appl. Mater. Interfaces.

[cit9] Zhou J., Fu S., Zhou S., Huang L., Wang C., Guan H., Pu D., Cui H., Wang C., Wang T., Meng W., Fang G., Ke W. (2024). Nat. Commun..

[cit10] Zhang T., Li M., Li X., Jiang X., Tao Y., Zheng S., Gu J., Zheng N., Bai G., Zhang M., Li C., Guan Y., Wang B., Fu Y. (2025). J. Am. Chem. Soc..

[cit11] Muddam R. S., Wang S., Maria Joseph Raj N. P., Wang Q., Wijesinghe P., Payne J., Dyer M. S., Bowen C., Krishnan Jagadamma L. (2025). Adv. Energy Mater..

[cit12] Mączka M., Zaręba J. K., Gągor A., Stefańska D., Ptak M., Roleder K., Kajewski D., Soszyński A., Fedoruk K., Sieradzki A. (2021). Chem. Mater..

[cit13] Mączka M., Sobczak S., Ratajczyk P., Leite F. F., Paraguassu W., Dybała F., Herman A. P., Kudrawiec R., Katrusiak A. (2022). Chem. Mater..

[cit14] Landrigan P. J. (1990). Environ. Health Perspect..

[cit15] Babayigit A., Ethirajan A., Muller M., Conings B. (2016). Nat. Mater..

[cit16] Ni H.-F., Zhou Q.-F., Luo J.-Q., Teri G., Pan L., Ye L.-K., Jia Q.-Q., Huang P.-Z., Liu P.-G., Wang C.-F., Zhang Z.-X., Fu D.-W., Zhang Y. (2025). Nat. Commun..

[cit17] Li X., Yue Z., Zhang F., Wang Q., Wei Q., Sun Z., Luo J., Liu X. (2024). Adv. Energy Mater..

[cit18] Sun P.-P., Kripalani D. R., Chi W., Snyder S. A., Zhou K. (2021). Mater. Today.

[cit19] Liang L., Gao P. (2018). Adv. Sci..

[cit20] Zhang Z.-X., Wang H., Ni H.-F., Wang N., Wang C.-F., Huang P.-Z., Jia Q.-Q., Teri G., Fu D.-W., Zhang Y., An Z., Zhang Y. (2024). Angew. Chem., Int. Ed..

[cit21] Rok M., Prytys J. K., Kinzhybalo V., Bator G. (2017). Dalton Trans..

[cit22] Hu S.-Q., Li M.-Z., Chen Z.-H., Zhou J.-S., Ji L.-Y., Ai Y., Chen X.-G. (2024). Inorg. Chem. Front..

[cit23] Rok M., Moskwa M., Działowa M., Bieńko A., Rajnák C., Boča R., Bator G. (2019). Dalton Trans..

[cit24] Rok M., Bator G., Zarychta B., Dziuk B., Repeć J., Medycki W., Zamponi M., Usevičius G., Šimėnas M., Banys J. (2019). Dalton Trans..

[cit25] Igbari F., Wang Z.-K., Liao L.-S. (2019). Adv. Energy Mater..

[cit26] Shi C., Ma J.-J., Jiang J.-Y., Hua M.-M., Xu Q., Yu H., Zhang Y., Ye H.-Y. (2020). J. Am. Chem. Soc..

[cit27] Wang C.-F., Yang Y., Hu Y., Ma C., Ni H.-F., Liu P.-G., Lu H.-F., Zhang Z.-X., Wang J., Zhang Y., Fu D.-W., Zhao K., Zhang Y. (2024). Angew. Chem., Int. Ed..

[cit28] Xiao Z., Song Z., Yan Y. (2019). Adv. Mater..

[cit29] Han Z., Guan Q., Ye H., Liu Y., Wu J., Xu L., Zhang C., Li H., Yin Q., Luo J. (2025). Inorg. Chem. Front..

[cit30] Liu Y., Ma Y., Zeng X., Xu H., Guo W., Wang B., Hua L., Tang L., Luo J., Sun Z. (2023). Nat. Commun..

[cit31] Xu W.-J., Zelenovskii P., Tselev A., Verissimo L., Romanyuk K., Yuan W., Zhang W.-X., Kholkin A., Rocha J. (2023). Chem. Commun..

[cit32] Lv H.-P., Hu S.-Q., Bai Y.-J., Zhou J.-S., Ji L.-Y., Wang Z.-X., Ai Y., Qin Y., Chen X.-G. (2025). Chem. Sci..

[cit33] Mandal A., Gupta S., Dutta S., Pati S. K., Bhattacharyya S. (2023). Chem. Sci..

[cit34] Aizu K. (1969). J. Phys. Soc. Jpn..

[cit35] Khan A. I., Marti X., Serrao C., Ramesh R., Salahuddin S. (2015). Nano Lett..

[cit36] Zhang H.-Y., Hu C.-L., Hu Z.-B., Mao J.-G., Song Y., Xiong R.-G. (2020). J. Am. Chem. Soc..

[cit37] Li J., Zhu Y., Huang P.-Z., Fu D.-W., Jia Q.-Q., Lu H.-F. (2022). Chem.–Eur. J..

[cit38] Engel E. R., Takamizawa S. (2018). Angew. Chem., Int. Ed..

[cit39] Hu Y., You L., Xu B., Li T., Morris S. A., Li Y., Zhang Y., Wang X., Lee P. S., Fan H. J., Wang J. (2021). Nat. Mater..

[cit40] Connor B. A., Su A. C., Slavney A. H., Leppert L., Karunadasa H. I. (2023). Chem. Sci..

[cit41] Shi C., Ye L., Gong Z.-X., Ma J.-J., Wang Q.-W., Jiang J.-Y., Hua M.-M., Wang C.-F., Yu H., Zhang Y., Ye H.-Y. (2020). J. Am. Chem. Soc..

[cit42] Wolf F., Chau T., Han D., Spooner K. B., Righetto M., Doerflinger P., Wang S., Guntermann R., Hooijer R., Scanlon D. O., Ebert H., Dyakonov V., Herz L. M., Bein T. (2025). J. Am. Chem. Soc..

[cit43] Maçzka M. a., Ptak M., Ga̧gor A., Stefańska D., Zarȩba J. K., Sieradzki A. (2020). Chem. Mater..

[cit44] Song X., Liu X., Zhang D., Liao J., Zhu S., Zheng W. (2024). J. Am. Chem. Soc..

[cit45] Huang C.-R., Luo X., Chen X.-G., Song X.-J., Zhang Z.-X., Xiong R.-G. (2020). Natl. Sci. Rev..

[cit46] Pareja-Rivera C., Solis-Ibarra D. (2021). Adv. Opt. Mater..

[cit47] Ning W., Zhao X.-G., Klarbring J., Bai S., Ji F., Wang F., Simak S. I., Tao Y., Ren X.-M., Zhang L., Huang W., Abrikosov I. A., Gao F. (2019). Adv. Funct. Mater..

[cit48] Ji F., Klarbring J., Zhang B., Wang F., Wang L., Miao X., Ning W., Zhang M., Cai X., Bakhit B., Magnuson M., Ren X., Sun L., Fahlman M., Buyanova I. A., Chen W. M., Simak S. I., Abrikosov I. A., Gao F. (2024). Adv. Opt. Mater..

[cit49] Lassoued M. S., Wang T., Faizan A., Li Q.-W., Chen W.-P., Zheng Y.-Z. (2022). J. Mater. Chem. C.

[cit50] Su C.-Y., Yao Y.-F., Zhang Z.-X., Wang Y., Chen M., Huang P.-Z., Zhang Y., Qiao W.-C., Fu D.-W. (2022). Chem. Sci..

[cit51] Aizu K. (1970). J. Phys. Soc. Jpn..

[cit52] Sapriel J. (1975). Phys. Rev. B.

[cit53] Schlenker J. L., Gibbs G., Boisen M. (1978). Acta Crystallogr. Sect. A Cryst. Phys. Diffr. Theor. Gen. Crystallogr..

[cit54] Carpenter M. A., Salje E. K., Graeme-Barber A. (1998). Eur. J. Mineral..

[cit55] Carpenter M. A., Salje E. K., Graeme-Barber A. (1998). Eur. J. Mineral..

[cit56] Wang Z.-X., Liao W.-Q., Ye H.-Y., Zhang Y. (2015). Dalton Trans..

[cit57] Gong Y., Chen X., Zhao B., Wang J., Zhang W., Chen X. (2023). Chin. Chem. Lett..

[cit58] Li X.-N., Li P.-F., Wang Z.-X., Shi P.-P., Tang Y.-Y., Ye H.-Y. (2017). Polyhedron.

[cit59] Martín-García B., Spirito D., Biffi G., Artyukhin S., Bonaccorso F., Krahne R. (2021). J. Phys. Chem. Lett..

[cit60] Zelewski S. J., Urban J. M., Surrente A., Maude D. K., Kuc A., Schade L., Johnson R. D., Dollmann M., Nayak P. K., Snaith H. J., Radaelli P., Kudrawiec R., Nicholas R. J., Plochocka P., Baranowski M. (2019). J. Mater. Chem. C.

[cit61] Steele J. A., Puech P., Keshavarz M., Yang R., Banerjee S., Debroye E., Kim C. W., Yuan H., Heo N. H., Vanacken J., Walsh A., Hofkens J., Roeffaers M. B. J. (2018). ACS Nano.

[cit62] Bian H., Zhang W., Zhang N., Chen G., Xu D., Zhao W., Xu X. (2025). Nano Res..

[cit63] Martín-García B., Spirito D., Lin M.-L., Leng Y.-C., Artyukhin S., Tan P.-H., Krahne R. (2022). Adv. Opt. Mater..

[cit64] Luo B., Liang D., Sun S., Xiao Y., Lian X., Li X., Li M.-D., Huang X.-C., Zhang J. Z. (2020). J. Phys. Chem. Lett..

[cit65] Ye H.-Y., Liao W.-Q., Hu C.-L., Zhang Y., You Y.-M., Mao J.-G., Li P.-F., Xiong R.-G. (2016). Adv. Mater..

[cit66] Long L., Huang Z., Xu Z.-K., Gan T., Qin Y., Chen Z., Wang Z.-X. (2024). Inorg. Chem. Front..

[cit67] Connor B. A., Leppert L., Smith M. D., Neaton J. B., Karunadasa H. I. (2018). J. Am. Chem. Soc..

[cit68] Mao L., Teicher S. M. L., Stoumpos C. C., Kennard R. M., DeCrescent R. A., Wu G., Schuller J. A., Chabinyc M. L., Cheetham A. K., Seshadri R. (2019). J. Am. Chem. Soc..

[cit69] (a) CCDC 2286670: Experimental Crystal Structure Determination, 2025, 10.5517/ccdc.csd.cc2grgk8

