# Supplementary material for: Two-dimensional lead-free double perovskite ferroelastics with dynamic thermochromism
Source: Chem Sci. 2025 Nov 3;16(48):23385–93. doi: 10.1039/d5sc05158d (PMC12598617; doi:10.1039/d5sc05158d)
Supplement: SC-016-D5SC05158D-s001 [file SC-016-D5SC05158D-s001.pdf]

Supplementary Information (SI) for Chemical Science.
This journal is © The Royal Society of Chemistry 2025

Supplementary information

Table of contents

Experimental Measurement Methods S3

Detailed Spontaneous Strain Calculations S5

Fig. S1 Hydrogen bonds of $(CBA)_2PbBr_4$ (a) and $(CBA)_4AgBiBr_8$ (b). S7

Fig. S2 The organic and inorganic components of $(CBA)_2PbBr_4$ in the ferroelastic (a-c) and paraelastic (d-f) phases. The basic stacking structure of $(CBA)_2PbBr_4$ in the ferroelastic (g) and paraelastic (h) phases. (i) Schematic diagram of the relationship between the crystal cell of $(CBA)_2PbBr_4$ in the ferroelastic and paraelastic phases. For the convenience of expression, ferroelastic phase is abbreviated as FP, and paraelastic phase is abbreviated as PP. S7

Fig. S3 The calculation model used by DPA^+ cations in $(DPA)_4AgBiBr_8$ at different rotation angles. S8

Fig. S4 The calculation model used by CBA^+ cations in $(CBA)_2PbBr_4$ at different rotation angles. S8

Fig. S5 The calculation model used by CBA^+ cations in $(CBA)_4AgBiBr_8$ at different rotation angles. S9

Fig. S6 The rotational energy barrier of cation in $(DPA)_4AgBiBr_8$ (a), $(CBA)_2PbBr_4$ (b) and $(CBA)_4AgBiBr_8$ (c). S9

Fig. S7 The ferroelastic domains and topography of $(CBA)_2PbBr_4$ before (a), (b) and after (c), (d) applying mechanical stress. S10

Fig. S8 The ferroelastic domains and topography of $(CBA)_4AgBiBr_8$ before (a), (b) and after (c), (d) applying mechanical stress. S10

Fig. S9 Reversible thermochromic behavior of $(CBA)_4AgBiBr_8$. S11

Fig. S10 The temperature-dependent UV-vis absorbance spectra of $(CBA)_4AgBiBr_8$. S11

Fig. S11 Raman spectra of $(CBA)_4AgBiBr_8$ from 100 cm^{-1} to 200 cm^{-1} durning heating (a) and cooling (b) processes. Two prominent peaks at 293 K (c) and 413 K (d). The changes in Raman spectra during heating (e) and cooling (f) processes. S12

Fig. S12 Partial density of states (PDOS) for H (a), C (b), N (c), Pb (d), and Br (e) in $(CBA)_2PbBr_4$. S13

Fig. S13 Partial density of states (PDOS) for H (a), C (b), N (c), Ag (d), Bi (e) and Br (f) in $(CBA)_4AgBiBr_8$. S13

Fig. S14 Measured and simulated powder X-ray diffraction patterns of $(CBA)_2PbBr_4$ (a) and $(CBA)_4AgBiBr_8$ (b). S14

Table S1. Crystal data and structure refinements for $(CBA)_2PbBr_4$. S15

Table S2. Crystal data and structure refinements for $(CBA)_4AgBiBr_8$. S15

Table S3. Selected bond lengths [Å] and bond angles [°] for $(CBA)_2PbBr_4$ at 283 K. S16

Table S4. Selected bond lengths [Å] and bond angles [°] for $(CBA)_4AgBiBr_8$ at 150 K. S16

Table S5. Selected bond lengths [Å] and bond angles [°] for $(CBA)_2PbBr_4$ at 388 K. S17

Table S6. Selected bond lengths [Å] and bond angles [°] for $(CBA)_4AgBiBr_8$ at 347 K. S17

Table S7. Structural and bandgap parameters comparison of Ruddlesden-Popper (RP) Pb-Br perovskites (n=1) and Ag&Bi-Br double perovskites (n=1). S18

Experimental Measurement Methods

Physical measurements. Differential scanning calorimetry (DSC), dielectric measurements, powder X-ray diffraction, variable-temperature single-crystal X-ray diffraction, UV-vis absorbance spectral measurements and electronic structure calculations were described in Supplementary information Information.

DSC Measurements.

DSC measurements of $(DPA)_4AgBiBr_8$ and $(CBA)_4AgBiBr_8$ were carried out in the temperature range from 300 to 400 K at a rate of 20 K min^{-1} using a PerkinElmer Diamond DSC instrument.

Dielectric Measurements.

Complex dielectric permittivities were measured with a TH2828A impedance analyzer. The samples were made with pressed-powder pellet for dielectric measurements. Silver conductive paste deposited on the plate surfaces of samples were used as top and bottom electrodes.

Powder X-ray Diffraction.

Powder X-ray diffraction (PXRD) data were measured using a Rigaku D/MAX 2000 PC X-ray diffraction system with Cu Kα radiation in the 2θ range of 5°–55° with a step size of 0.02°.

Variable-Temperature Single-Crystal X-ray Crystallography.

Variable-temperature single-crystal XRD data of $(DPA)_4AgBiBr_8$ and $(CBA)_4AgBiBr_8$ were collected using a Bruker APEX-II CCD with Mo Kα radiation (λ = 0.71073 Å) at different temperature, whose processing was disposed by the APEX3. Variable-temperature crystal structures were solved using a direct method and subsequent continuous Fourier synthesis. Subsequently, these crystals were refined by full-matrix leastsquares methods based on F2 using the SHELXTL software package. Eventually, in addition to the asymmetric units and packing shown in main body using Diamond and 3ds Max, other relevant crystallographic data and structure refinement are listed in Table S1 and S2 in supporting information.

UV-vis Absorbance Spectral Measurements.

Ultraviolet Spectral Analyses were conducted on a Shimadzu UV-2450

spectrophotometer from 300 to 800 nm at room temperature.

For temperature-dependent ultraviolet spectral measurements, a PerkinElmer Lambda 1050 spectrophotometer (USA) equipped with an in-situ heating device was used instead. The measurements were performed within a heating range of 300 K to 400 K, followed by natural cooling back to 300 K.

The optical band gap (E_g) was estimated from Tauc equation:

$$[\alpha hv]^{1/n} = A(hv - Eg)$$

where h is Planck constant, v is the frequency of vibration, A is the proportional constant, α is the absorption coefficient, and n represents the nature of the sample's transition, n = 1/2 for direct and n = 2 for indirect transition. The E_g can be obtained from a Tauc plot by plotting $[\alpha hv]^{1/n}$ against the energy in electron volt.

Raman Spectrum.

The crystal was tested at a laser wavelength of 532 nm using the Alpha 300 confocal micro-Raman spectroscopy produced by WITec in Germany, using a backscatter configuration. The temperature control system used throughout the entire testing process is the THMS600E thermal platform produced by Linkam Scientific Instruments in the UK.

Density Functional Theory Calculations.

Density functional theory (DFT) were performed with the Vienna Ab Initio Simulation Package (VASP)[1]. The Perdew−Burke−Ernzerhof(PBE)[2] functional and frozen-core all electron projector augmented wave (PAW)[3] model was employed. The cutoff energy was set to 500 eV. And the convergence criteria for the residual force and energy on each atom during structure relaxation were set to 0.01 eV/Å and 10^{-5} eV, respectively.

Reference:

1. G. Kresse and J. Furthmüller, *Comput. Mater. Sci.*, 1996, **6**, 15-50.
2. J. P. Perdew, M. Ernzerhof and K. Burke, *J. Chem. Phys.*, 1996, **105**, 9982.
3. P. E. Blöchl, *Phys. Rev. B*, 1994, **50**, 17953-17979.

Detailed Spontaneous Strain Calculations

1. Spontaneous Strain Tensor Derivation For $(CBA)_2PbBr_4$ (*mmm*F2/*m*)

1.1 Prototype and Ferroelastic Phase Lattice Parameter Alignment

The paraelastic prototype (388 K, *Cmce*) has a_{PP} = 29.24 Å, b_{PP} = 8.247 Å, c_{PP} = 8.249 Å (Z = 2), and the ferroelastic phase (283 K, $P2_1/c$) has a_{FP} = 14.339 Å, b_{FP} = 8.289 Å, c_{FP} = 8.232 Å, β_{FP} = 99.762° (Z = 2). The orthorhombic prototype's a_{PP} is twice the ferroelastic phase's a_{FP} (a result of orthorhombic-to-monoclinic symmetry breaking), so $a_{PP,norm} = a_{PP}/2$ = 14.62 Å is used for strain calculation.

1.2 Strain Component Calculation

Tensile strains: $\varepsilon_{11} = (a_{FP} - a_{PP,norm})/a_{PP,norm}$ = (14.339 - 14.62)/14.62 ≈ -0.0192 (contraction along a-axis); $\varepsilon_{22} = (b_{FP} - b_{PP})/b_{PP}$ = (8.289 - 8.247)/8.247 ≈ 0.0051 (expansion along b-axis); $\varepsilon_{33} = (c_{FP} - c_{PP})/c_{PP}$ = (8.232 - 8.249)/8.249 ≈ 0.0021 (contraction along c-axis).

Shear strain: For monoclinic crystals, shear strain ε_{12} arises from β deviation from 90°: $\varepsilon_{12} = \varepsilon_{21} = (1/2)\cos\beta_{FP}$ = (1/2)cos(99.762°) ≈ -0.0853.

Zero components: $\varepsilon_{13} = \varepsilon_{31} = \varepsilon_{23} = \varepsilon_{32} = 0$, as 2/*m* symmetry prohibits shear in *a*-*c* and *b*-*c* planes.

1.3 Spontaneous Strain Tensor

$$\varepsilon_{ij} = \begin{bmatrix} -0.0192 & -0.0853 & 0 \\ -0.0853 & 0.0051 & 0 \\ 0 & 0 & -0.0021 \end{bmatrix}$$

1.4 Total Spontaneous Strain

$$\varepsilon_{ss} = \sqrt{\varepsilon_{11}^2 + \varepsilon_{22}^2 + \varepsilon_{33}^2 + 2\varepsilon_{12}^2} = \sqrt{(-0.0192)^2 + 0.0051^2 + (-0.00\text{[illegible]}} \approx 0.124$$

2. Spontaneous Strain Tensor Derivation For $(CBA)_4AgBiBr_8$ (4/*mmm*F2/*m*)

2.1 Prototype Scaling for Z Consistency

The paraelastic prototype (347 K, *I*4/*mmm*) has $a_{PP} = b_{PP}$ = 5.818 Å, c_{PP} = 28.386 Å (Z = 1), while the ferroelastic phase (150 K, $P2_1/c$) has Z = 2. The tetragonal-to-monoclinic transitions require scaling a_{PP} by $\sqrt{2}$ to match Z = 2: $a_{PP,scaled} = a_{PP} \times \sqrt{2}$ =

$5.818 \times 1.414 \approx 8.23$ Å; $c_{\mathrm{PP,scaled}} = c_{\mathrm{PP}} = 28.386$ Å.

2.2 Strain Component Calculation

Tensile strains: $\varepsilon_{11} = (a_{\mathrm{FP}} - a_{\mathrm{PP,scaled}})/a_{\mathrm{PP,scaled}} = (8.1901 - 8.23)/8.23 \approx -0.0048$ (contraction along a-axis); $\varepsilon_{22} = (b_{\mathrm{FP}} - a_{\mathrm{PP,scaled}})/a_{\mathrm{PP,scaled}} = (8.2705 - 8.23)/8.23 \approx 0.005$ (expansion along b-axis); $\varepsilon_{33} = (c_{\mathrm{FP}} - c_{\mathrm{PP,scaled}})/c_{\mathrm{PP,scaled}} = (26.638 - 28.386)/28.386 \approx -0.0616$ (contraction along c-axis).

Shear strain: Shear strain ε_{13} arises from β deviation.

$\varepsilon_{13} = \varepsilon_{31} = (1/2)\cos\beta_{\mathrm{FP}} = (1/2)\cos(98.040°) \approx -0.0698$.

Zero components: $\varepsilon_{12} = \varepsilon_{21} = \varepsilon_{23} = \varepsilon_{32} = 0$, forbidden by $4/mmm\mathrm{F}2/m$ symmetry breaking.

2.3 Spontaneous Strain Tensor

$$\varepsilon_{ij} = \begin{bmatrix} -0.0048 & 0 & -0.0698 \\ 0 & 0.0050 & 0 \\ -0.0698 & 0 & -0.0616 \end{bmatrix}$$

2.4 Total Spontaneous Strain

$$\varepsilon_{ss} = \sqrt{\varepsilon_{11}^2 + \varepsilon_{22}^2 + \varepsilon_{33}^2 + 2\varepsilon_{13}^2} = \sqrt{(-0.048)^2 + 0.0050^2 + (-0.061 \text{[illegible]}} \approx 0.131$$

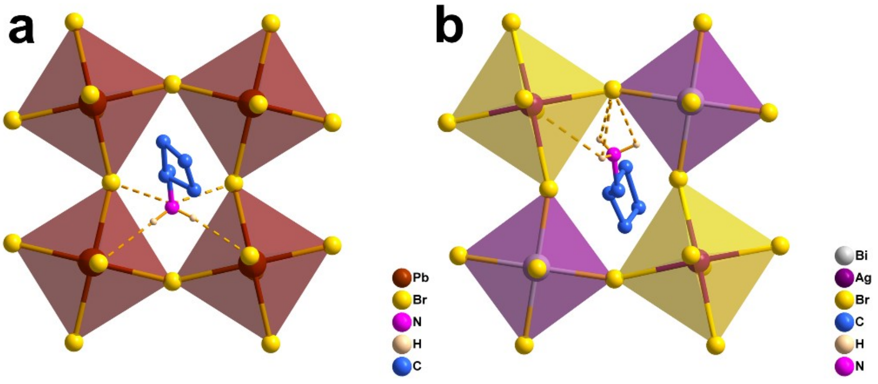

Fig. S1 Hydrogen bonds of $(CBA)_2PbBr_4$ (a) and $(CBA)_4AgBiBr_8$ (b).

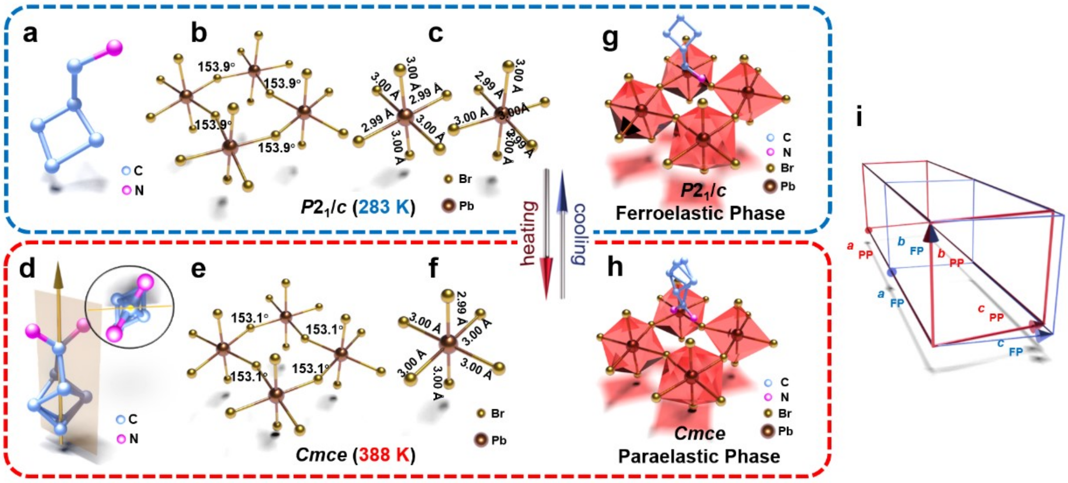

Fig. S2 The organic and inorganic components of $(CBA)_2PbBr_4$ in the ferroelastic (a-c) and paraelastic (d-f) phases. The basic stacking structure of $(CBA)_2PbBr_4$ in the ferroelastic (g) and paraelastic (h) phases. (i) Schematic diagram of the relationship between the crystal cell of $(CBA)_2PbBr_4$ in the ferroelastic and paraelastic phases. For the convenience of expression, ferroelastic phase is abbreviated as FP, and paraelastic phase is abbreviated as PP.

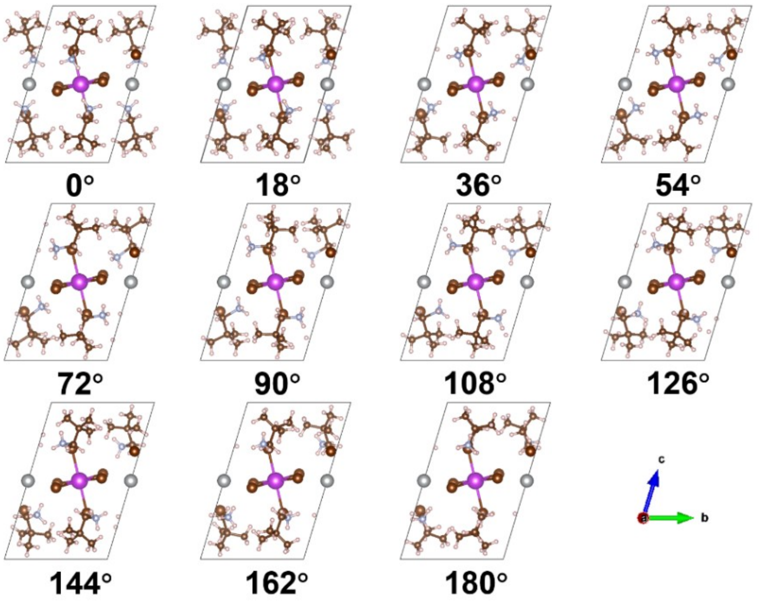

Fig. S3 The calculation model used by DPA^+ cations in $(DPA)_4AgBiBr_8$ at different rotation angles.

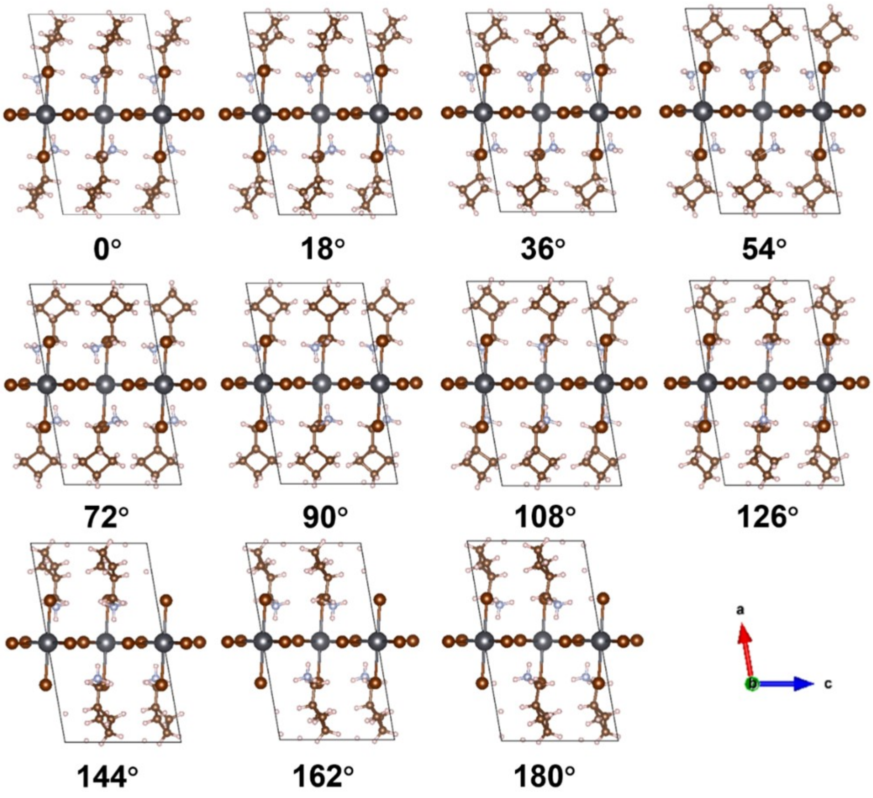

Fig. S4 The calculation model used by CBA^+ cations in $(CBA)_2PbBr_4$ at different rotation angles.

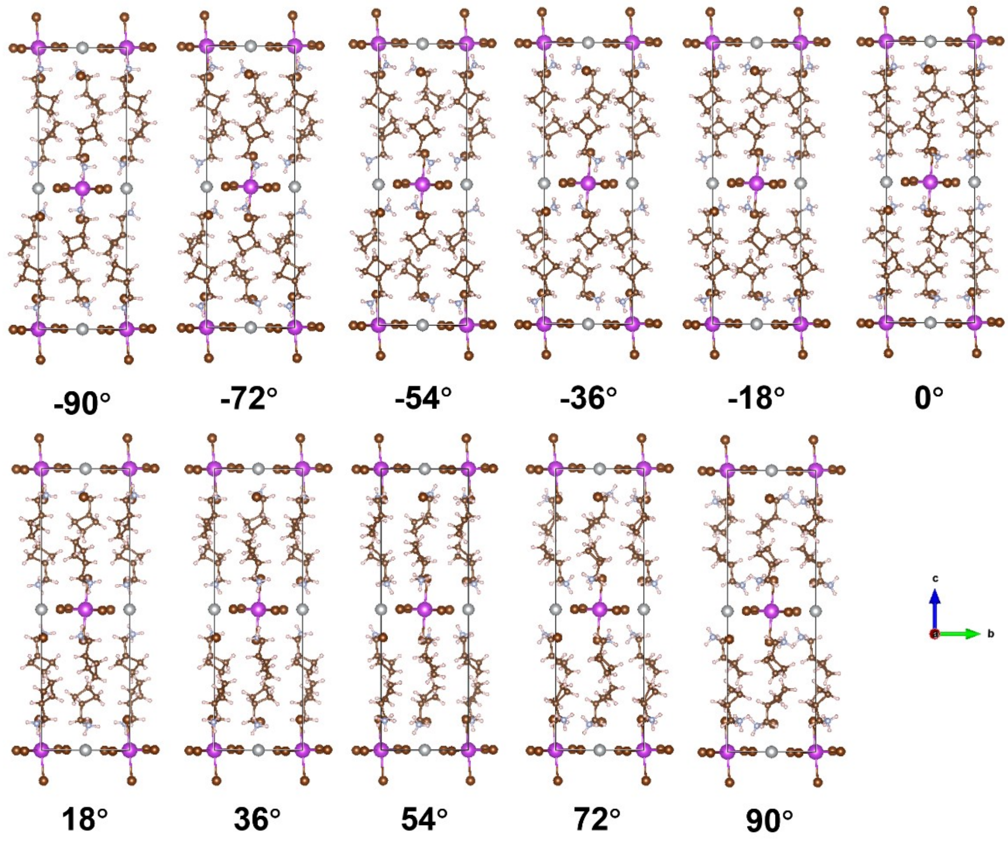

Fig. S5 The calculation model used by CBA^+ cations in $(CBA)_4AgBiBr_8$ at different rotation angles.

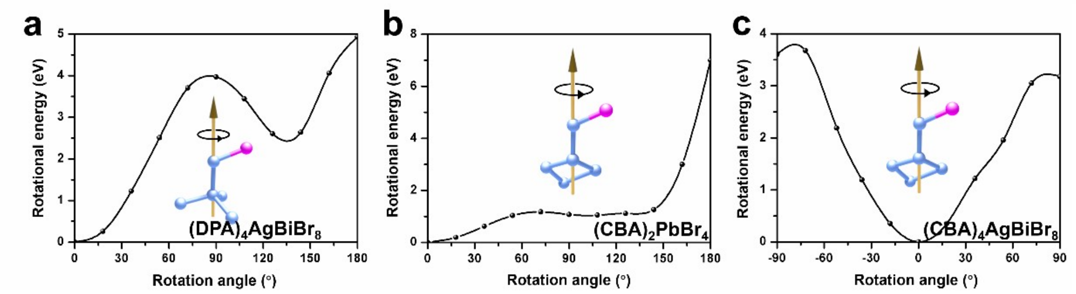

Fig. S6 The rotational energy barrier of cation in $(DPA)_4AgBiBr_8$ (a), $(CBA)_2PbBr_4$ (b) and $(CBA)_4AgBiBr_8$ (c).

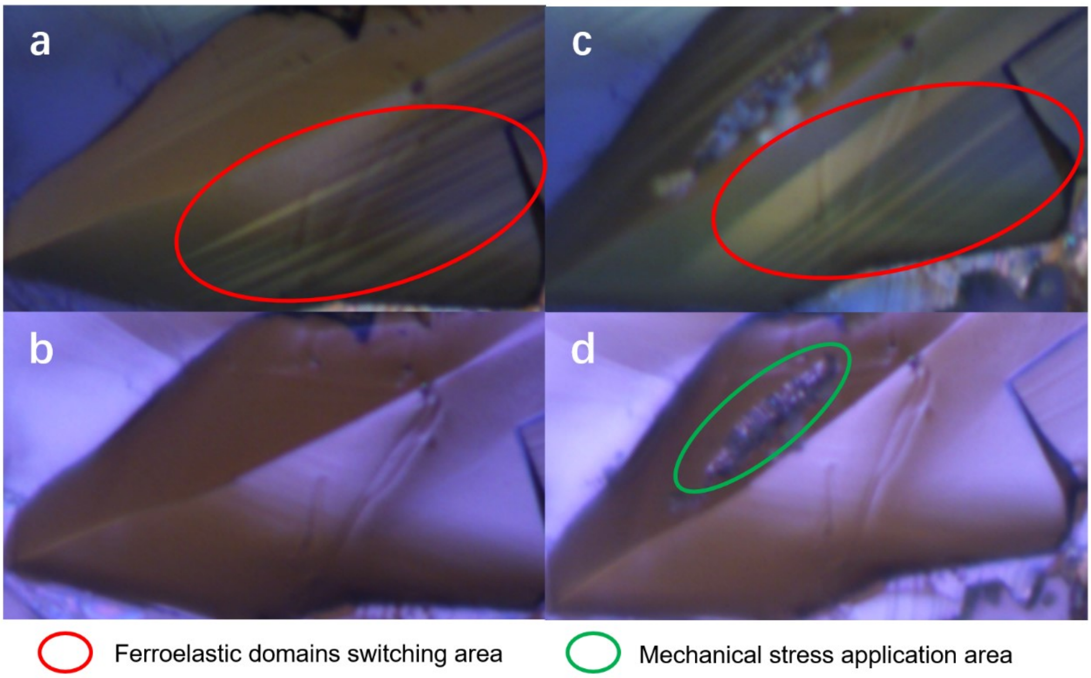

Fig. S7 The ferroelastic domains and topography of $(CBA)_2PbBr_4$ before (a), (b) and after (c), (d) applying mechanical stress.

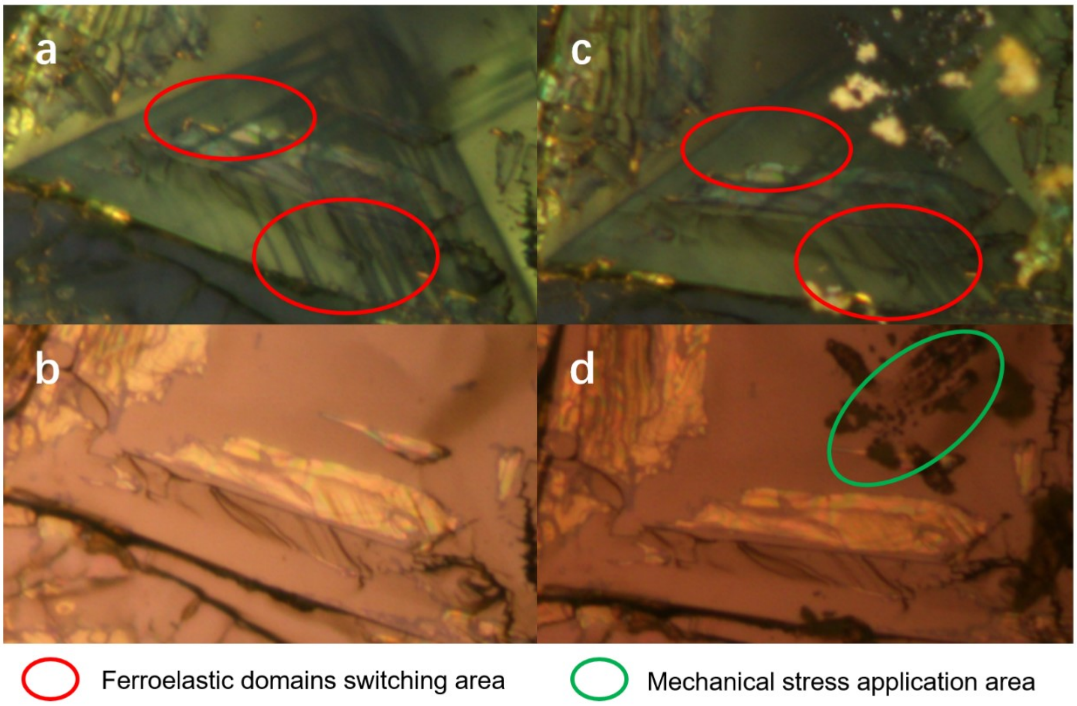

Fig. S8 The ferroelastic domains and topography of $(CBA)_4AgBiBr_8$ before (a), (b) and after (c), (d) applying mechanical stress.

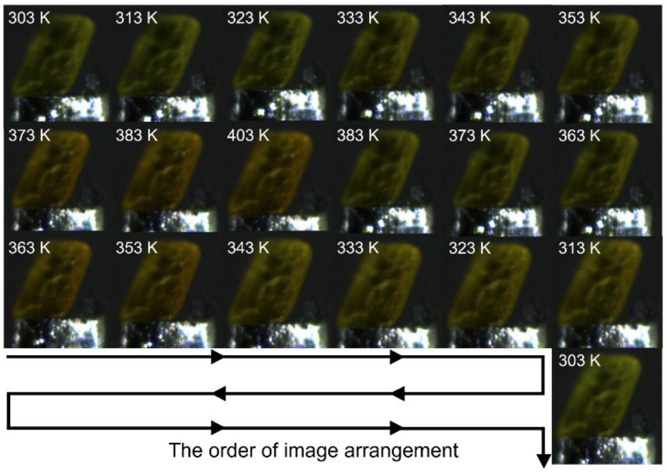

Fig. S9 Reversible thermochromic behavior of $(CBA)_4AgBiBr_8$.

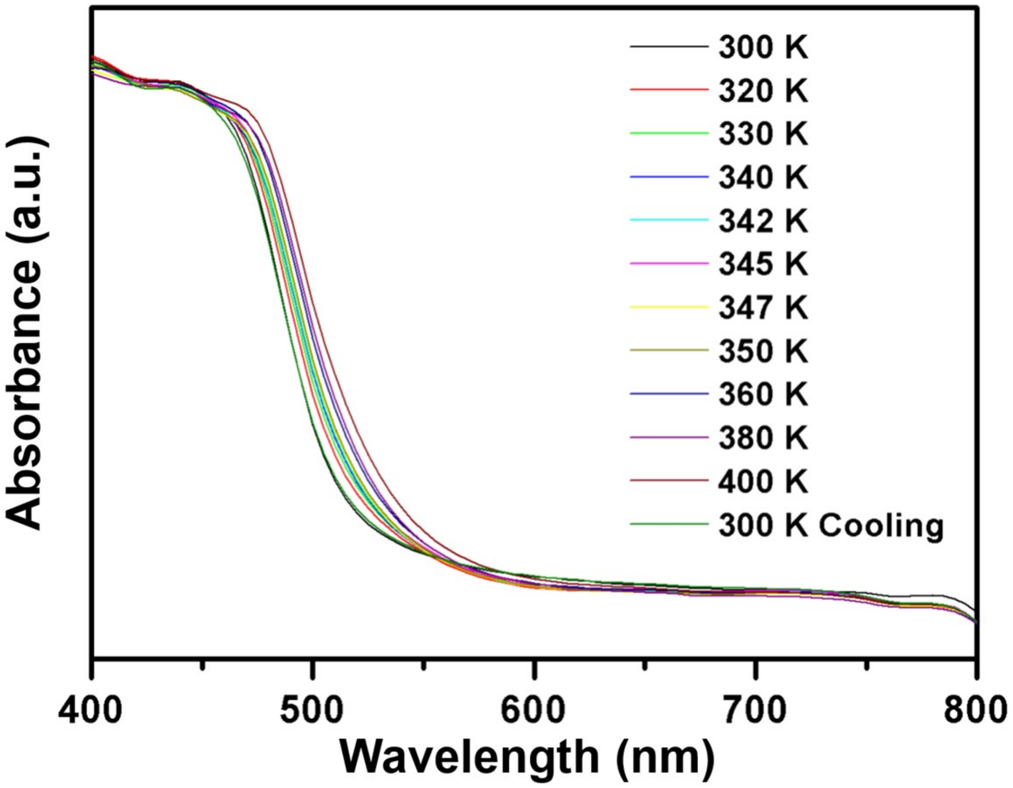

Fig. S10 The temperature-dependent UV-vis absorbance spectra of $(CBA)_4AgBiBr_8$.

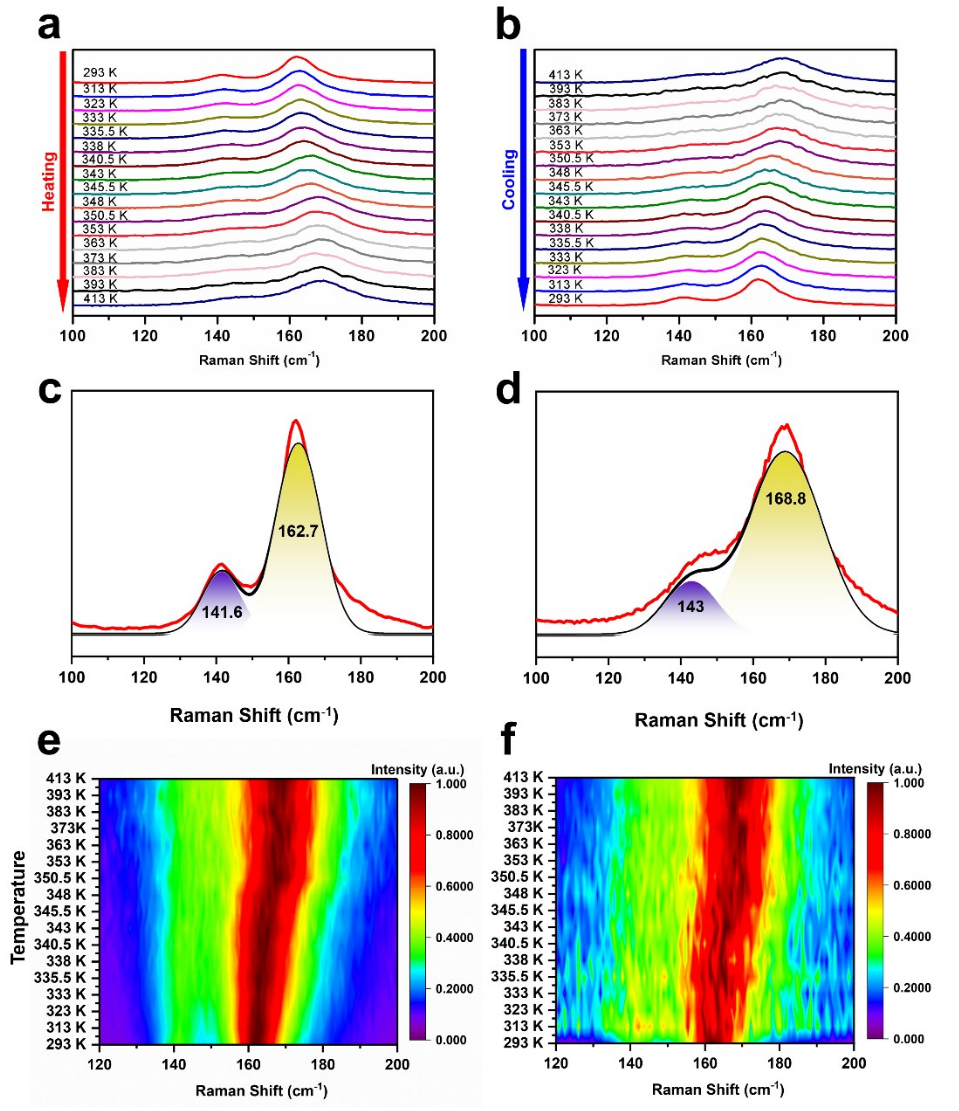

Fig. S11 Raman spectra of $(CBA)_4AgBiBr_8$ from 100 cm^{-1} to 200 cm^{-1} durning heating (a) and cooling (b) processes. Two prominent peaks at 293 K (c) and 413 K (d). The changes in Raman spectra during heating (e) and cooling (f) processes.

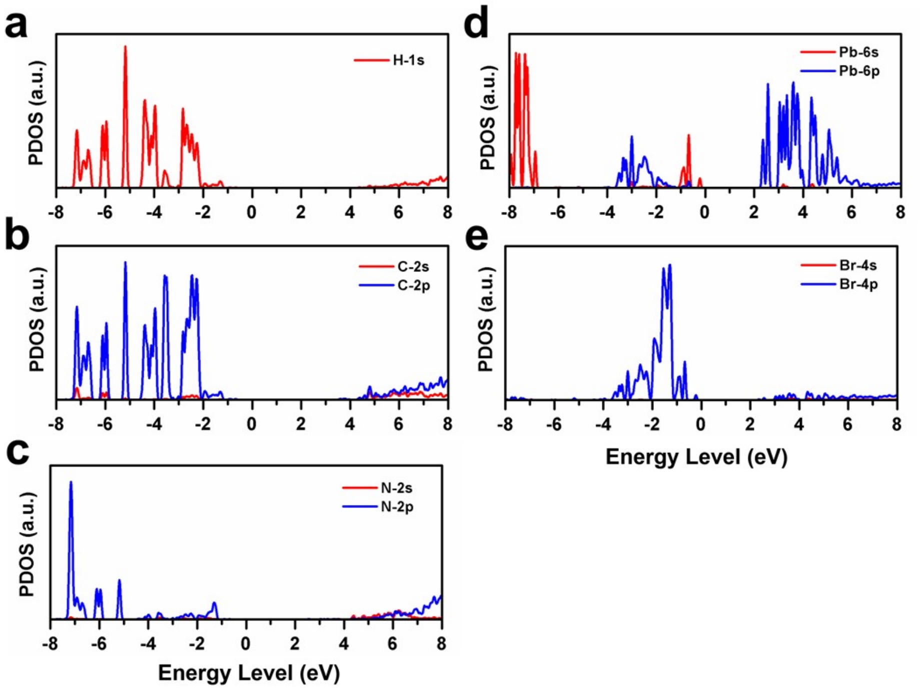

Fig. S12 Partial density of states (PDOS) for H (a), C (b), N (c), Pb (d), and Br (e) in $(CBA)_2PbBr_4$.

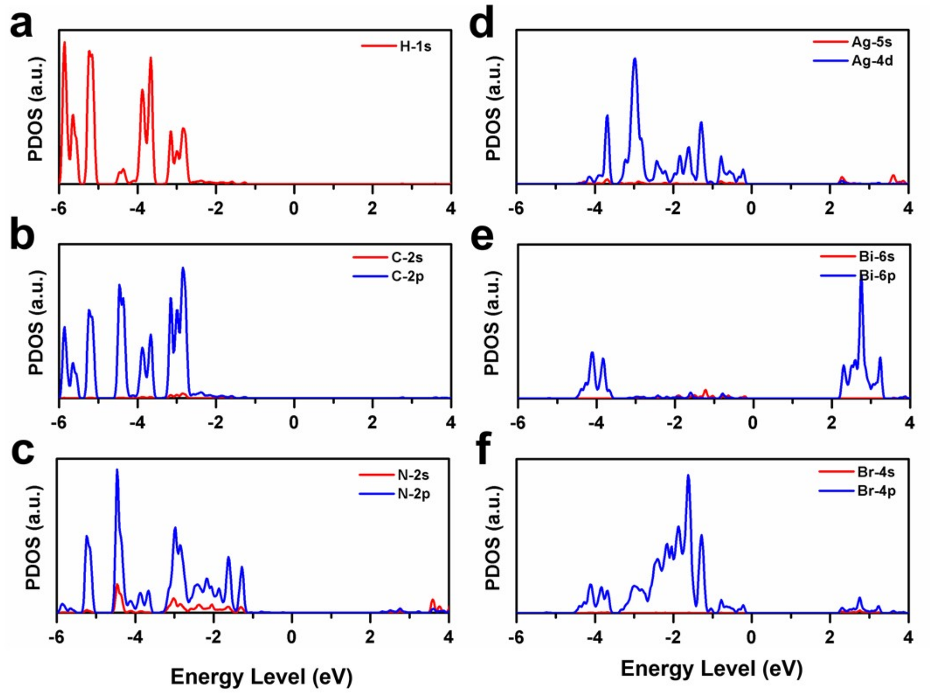

Fig. S13 Partial density of states (PDOS) for H (a), C (b), N (c), Ag (d), Bi (e) and Br (f) in $(CBA)_4AgBiBr_8$.

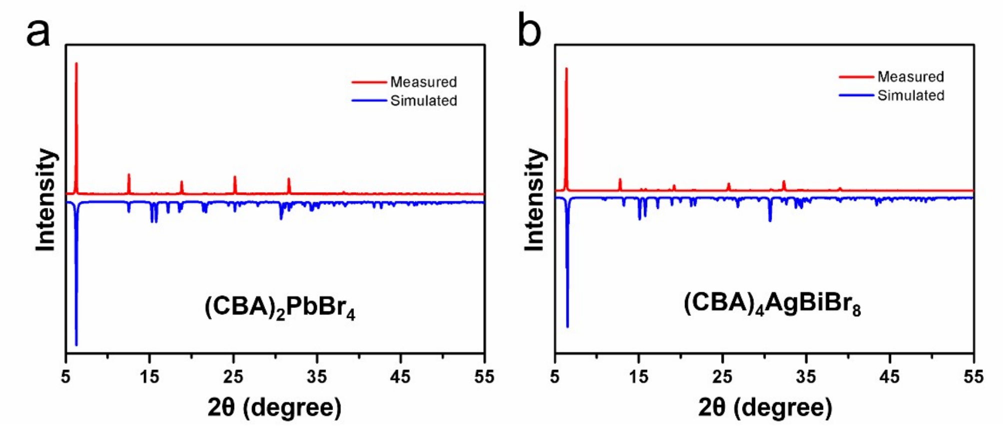

Fig. S14 Measured and simulated powder X-ray diffraction patterns of $(CBA)_2PbBr_4$ (a) and $(CBA)_4AgBiBr_8$ (b).

Table S1. Crystal data and structure refinements for **$(CBA)_2PbBr_4$.**

	283 K	388 K
Empirical formula	$C_{10}H_{24}PbBr_4N_2$	$C_{20}Pb_2Br_8N_4$
Formula weight	699.14	1349.90
Crystal system	Monoclinic	Orthorhombic
Space group	$P2_1/c$	*Cmce*
a (Å)	14.339(7)	29.24(3)
b (Å)	8.289(3)	8.247(6)
c (Å)	8.232(3)	8.249(6)
α (°)	90	90
β (°)	99.762(12)	90
γ (°)	90	90
Volume(Å^3)	964.3(7)	1989(3)
Z	2	2
Radiation type	MoK\a	MoK\a
Absorption correction	Multi-scan	Multi-scan
D_{calc}/g cm^{-3}	2.408	2.254
F (000)	640	1184
GOF	1.017	1.13
$R_1[I > 2\sigma(I)]$	0.045	0.046
w$R2[I > 2\sigma(I)]$	0.120	0.119

Table S2. Crystal data and structure refinements for **$(CBA)_4AgBiBr_8$.**

	150 K	347 K
Empirical formula	$C_{20}H_{48}AgBiBr_8N_4$	$C_{20}AgBiBr_8N_4$
Formula weight	1300.75	1252.37
Crystal system	Monoclinic	Tetragonal
Space group	$P2_1/c$	*I*4/*mmm*
a (Å)	8.1901(14)	5.818(2)
b (Å)	8.2705(14)	5.818(2)
c (Å)	26.638(5)	28.386 (15)
α (°)	90	90
β (°)	98.040(6)	90
γ (°)	90	90
Volume(Å^3)	1786.6(5)	960.8 (8)
Z	2	1
Radiation type	MoK\a	MoK\a
Absorption correction	Multi-scan	Multi-scan
D_{calc}/g cm^{-3}	2.418	2.164
F (000)	1212	558
GOF	1.253	1.05

$R_1[I > 2\sigma(I)]$	0.113	0.061
$wR2[I > 2\sigma(I)]$	0.297	0.183

Table S3. Selected bond lengths [Å] and bond angles [°] for **$(CBA)_2PbBr_4$** at 283 K.

Temperature	bond lengths [Å]		bond angles [°]	
283 K	Pb01—$Br02^{i}$	2.9942 (10)	$Br02^{i}$—Pb01—Br02	180.0
	Pb01—$Br02^{ii}$	3.0026 (10)	Br02—Pb01—$Br02^{ii}$	90.37 (3)
	Pb01—Br02	2.9942 (10)	$Br02^{i}$—Pb01—$Br02^{ii}$	89.63 (3)
	Pb01—$Br02^{iii}$	3.0026 (10)	$Br02^{i}$—Pb01—$Br02^{iii}$	90.37 (3)
	Pb01—$Br03^{i}$	3.0030 (16)	Br02—Pb01—$Br02^{iii}$	89.63 (3)
	Pb01—Br03	3.0030 (16)	$Br02^{iii}$—Pb01—$Br02^{ii}$	180.0
			$Br02^{iii}$—Pb01—$Br03^{i}$	94.51 (2)
			$Br02^{i}$—Pb01—$Br03^{i}$	90.96 (2)
			$Br02^{ii}$—Pb01—Br03	94.51 (2)
			Br02—Pb01—Br03	90.95 (2)
			Br02—Pb01—$Br03^{i}$	89.05 (2)
			$Br02^{iii}$—Pb01—Br03	85.49 (2)
			$Br02^{i}$—Pb01—Br03	89.04 (2)
			$Br02^{ii}$—Pb01—$Br03^{i}$	85.49 (2)
			Br03—Pb01—$Br03^{i}$	180.0
			Pb01—Br02—$Pb01^{iv}$	153.85 (2)

Symmetry codes: (i) $-x+1, -y+1, -z+1$; (ii) $-x+1, y+1/2, -z+1/2$; (iii) $x, -y+1/2, z+1/2$; (iv) $-x+1, y-1/2, -z+1/2$.

Table S4. Selected bond lengths [Å] and bond angles [°] for **$(CBA)_4AgBiBr_8$** at 150 K.

Temperature	bond lengths [Å]		bond angles [°]	
150 K	Bi1—$Br1^{i}$	2.8474 (8)	$Br1^{i}$—Bi1—Br1	180.0
	Bi1—Br1	2.8474 (8)	Br1—Bi1—Br2	93.69 (2)
	Bi1—$Br2^{i}$	2.8774 (9)	$Br1^{i}$—Bi1—$Br2^{i}$	93.69 (2)
	Bi1—Br2	2.8773 (9)	Br1—Bi1—$Br2^{i}$	86.31 (2)
	Bi1—Br4	2.8445 (8)	$Br1^{i}$—Bi1—Br2	86.31 (2)
	Bi1—$Br4^{i}$	2.8445 (8)	Br2—Bi1—$Br2^{i}$	180.0
	Ag1—$Br1^{ii}$	3.0596 (8)	$Br4^{i}$—Bi1—$Br1^{i}$	89.44 (3)
	Ag1—Br1	3.0596 (8)	Br4—Bi1—Br1	89.44 (3)
	Ag1—$Br6^{ii}$	2.6512 (10)	Br4—Bi1—$Br1^{i}$	90.56 (3)
	Ag1—Br6	2.6512 (10)	$Br4^{i}$—Bi1—Br1	90.56 (3)
			$Br4^{i}$—Bi1—Br2	89.24 (2)

Br4—Bi1—Br2^{i}	89.25 (2)
Br4—Bi1—Br2	90.75 (2)
Br4^{i}—Bi1—Br2^{i}	90.76 (2)
Br4^{i}—Bi1—Br4	179.999 (16)
Br1—Ag1—Br1^{ii}	180.0
Br6—Ag1—Br1	90.64 (2)
Br6^{ii}—Ag1—Br1^{ii}	90.64 (2)
Br6—Ag1—Br1^{ii}	89.36 (2)
Br6^{ii}—Ag1—Br1	89.36 (2)
Br6—Ag1—Br6^{ii}	180.0
Bi1—Br1—Ag1	160.27 (3)

Symmetry codes: (i) −x+1, −y+1, −z+1; (ii) −x+2, −y, −z+1.

Table S5. Selected bond lengths [Å] and bond angles [°] for **$(CBA)_2PbBr_4$** at 388 K.

Temperature	bond lengths [Å]		bond angles [°]	
388 K	Pb01—Br02^{i}	2.9981 (19)	Br02^{iii}—Pb01—Br02^{ii}	180.00 (3)
	Pb01—Br02	2.9981 (19)	Br02^{iii}—Pb01—Br02^{i}	90.01 (5)
	Pb01—Br02^{ii}	2.9978 (18)	Br02^{ii}—Pb01—Br02	90.01 (5)
	Pb01—Br02^{iii}	2.9978 (18)	Br02^{ii}—Pb01—Br02^{i}	89.99 (5)
	Pb01—Br03^{i}	2.989 (3)	Br02^{iii}—Pb01—Br02	89.99 (5)
	Pb01—Br03	2.989 (3)	Br02—Pb01—Br02^{i}	180.0
			Br03—Pb01—Br02^{iii}	90.0
			Br03—Pb01—Br02^{i}	90.0
			Br03^{i}—Pb01—Br02	90.0
			Br03^{i}—Pb01—Br02^{ii}	90.0
			Br03^{i}—Pb01—Br02^{iii}	90.0
			Br03—Pb01—Br02^{ii}	90.0
			Br03^{i}—Pb01—Br02^{i}	90.0
			Br03—Pb01—Br02	90.0
			Br03^{i}—Pb01—Br03	180.0
			Pb01^{v}—Br02—Pb01	153.14 (4)

Symmetry codes: (i) −x+1, −y+1, −z+1; (ii) −x+1, −y+3/2, z+1/2; (iii) x, y−1/2, −z+1/2; (iv) x, −y+1, −z; (v) −x+1, −y+3/2, z−1/2.

Table S6. Selected bond lengths [Å] and bond angles [°] for **$(CBA)_4AgBiBr_8$** at 347 K.

Temperature	bond lengths [Å]		bond angles [°]	
347 K	Ag/Bi1—Br1^{viii}	2.731 (3)	Br1^{viii}—Ag/Bi1—Br1	180.0

Ag/Bi1—Br1	2.731 (3)	Br1^{viii}—Ag/Bi1—Br2^{ix}	90.0
Ag/Bi1—Br2^{ix}	2.9090 (10)	Br1—Ag/Bi1—Br2^{ix}	90.0
Ag/Bi1—Br2^{iv}	2.9090 (10)	Br1^{viii}—Ag/Bi1—Br2^{iv}	90.0
Ag/Bi1—Br2	2.9090 (10)	Br1—Ag/Bi1—Br2^{iv}	90.0
Ag/Bi1—Br2^{x}	2.9090 (10)	Br2^{ix}—Ag/Bi1—Br2^{iv}	180.0
		Br1^{viii}—Ag/Bi1—Br2	90.0
		Br1—Ag/Bi1—Br2	90.0
		Br2^{ix}—Ag/Bi1—Br2	90.0
		Br2^{iv}—Ag/Bi1—Br2	90.0
		Br1^{viii}—Ag/Bi1—Br2^{x}	90.0
		Br1—Ag/Bi1—Br2^{x}	90.0
		Br2^{ix}—Ag/Bi1—Br2^{x}	90.0
		Br2^{iv}—Ag/Bi1—Br2^{x}	90.0
		Br2—Ag/Bi1—Br2^{x}	180.0
		Ag/Bi1^{xi}—Br2—Ag/Bi1	180.0

Symmetry codes: (iv) −y+3, x, z; (viii) −x+2, −y+4, −z+1; (ix) −y+3, x+1, z; (x) x−1, y, z; (xi) x+1, y, z.

Table S7. Structural and bandgap parameters comparison of Ruddlesden-Popper (RP) Pb-Br perovskites (n=1) and Ag&Bi-Br double perovskites (n=1).

Materials	Dist. between layers (Å)	out-of-plane tilting Br-X-Br angle (θ_{out}, °) and D_{out}	in-plane tilting X-Br-X angle (θ_{in}, °) and D_{in}	Band gap (eV)	CCDC	Ref.
$PA_4AgBiBr_8$	12.2515	180 and 0	163.8 and 16.2	2.41	1968516	1
(3-bromopropyl aminium)$_4$AgBiBr$_8$	12.996	$(AgBr_6)^{5-}$ 169.5 and 10.5 $(BiBr_6)^{3-}$ 173.8 and 6.2	162.9 and 17.1<br>164.6 and 15.4	2.52	2224043	2
**(CBA)$_4$AgBiBr$_8$**	**13.233**	**180 and 0**	**160.3 and 19.7**	**2.22**	**2270990**	**This work**
(DPA)$_4$AgBiBr$_8$	13.349	180 and 0	157.4 and 22.6<br>169.7 and 10.3	2.44	2115543	3
(BA)$_4$AgBiBr$_8$	13.5225	180 and 0	166.8 and 13.2	2.61	1814798	4-5
(FPEA)$_4$AgBiBr$_8$	16.2199	180 and 0	160.7 and 19.3	2.39	2151234	4, 6
(OcA)$_4$AgBiBr$_8$	20.7198	180 and 0	169.5 and 10.5	2.45	1968519	1
$Cs_2AgBiBr_6$	/	180 and 0	180 and 0	2.19	/	7
MHy_2PbBr_4	8.909	180 and 0	167.0 and 13,<br>175.3 and 4.7	3.02	2018799	8

[4,4-DFPD]$_2$PbBr$_4$	12.62	167.5 and 12.5<br>166.1 and 13.9<br>168.0 and 12	177.1 and 2.9<br>171.2 and 8.8<br>180 and 0	2.95	2195164	9
CHA$_2$PbBr$_4$	13.999	165.1 and 14.9	148.6 and 22<br>158.0 and 31.4	3.05	1409218	10
**CBA$_2$PbBr$_4$**	**14.131**	**180 and 0**	**153.9 and 26.1**	**2.97**	**2286670**	**This work**
(R-MPA)(BrEA)PbBr$_4$	15.3717	177.8 and 2.2	148.3 and 31.7<br>152.3 and 27.7	2.98	2315904	11
(PEA)$_2$PbBr$_4$	16.5122	179.4 and 0.6<br>179.5 and 0.5	152.2 and 27.8<br>151.1 and 28.9	3.0	1903529	12
($C_8H_{11}FN$)$_2$PbBr$_4$	16.6431	180 and 0	154.7 and 25.3	2.88	2006717	13
(R/S-FMBA)$_2$PbBr$_4$	16.6747	166.9 and 13.1	157.0 and 23<br>142.3 and 37.7	2.96	2252841,<br>2252843	14
BZA$_2$PbBr$_4$	16.6764	179.0 and 1.0	149.9 and 30.1	2.92	1879417	12
R-/S-[3MeOPEA]$_2$PbBr$_4$	/	/	/	3.0	/	15
R-/S-(BrBA)$_2$PbBr$_4$	/	/	/	2.93/2.94	/	16

Abbreviations: CBA=cyclobutylmethanaminium, OcA=octylammonium, PA=propylammonium, DPA=2,2-dimethylpropan-1-aminium, FPEA=fluorophenethylammonium, BA=butan-1-aminium, MHy=$CH_3NH_2NH_2^+$, CHA=cyclohexylammonium, BZA=benzylammonium, PEA= $C_6H_5C_2H_4NH_3^+$, 4,4-DFPD=4,4-difluoropiperidinylaminium， FMBA=4-fluorophenethylamine, R-MPA = methylphenethylamm-onium; BrEA = 2-bromoethylamine, R-/S-[3MeOPEA]=R-/S-1-(3-Methoxyphenyl, R-/S-(BrBA)= R-/S-4-bromobutan-2-aminium

Reference:

(1) Mao, L.; Teicher, S. M. L.; Stoumpos, C. C.; Kennard, R. M.; DeCrescent, R. A.; Wu, G.; Schuller, J. A.; Chabinyc, M. L.; Cheetham, A. K.; Seshadri, R. Chemical and Structural Diversity of Hybrid Layered Double Perovskite Halides. *J. Am. Chem. Soc.* **2019**, *141*, 19099-19109.

(2) Yue, Z.; Wu, F.; Li, X.; Liu, Y.; Luo, J.; Liu, X. Centimeter-sized single crystal of a lead-free halide double perovskite with ferroelastic phase transition-triggered switchable dielectric properties. *Sci. China Mater.* **2023**, *66*, 3977-3983.

(3) Su, C.-Y.; Yao, Y.-F.; Zhang, Z.-X.; Wang, Y.; Chen, M.; Huang, P.-Z.; Zhang,

Y.; Qiao, W.-C.; Fu, D.-W. The construction of a two-dimensional organic-inorganic hybrid double perovskite ferroelastic with high T_c and narrow band gap. *Chem. Sci.* **2022**, *13*, 4794-4800.
(4) Ge, M.; Chen, S.; Fu, X.; Feng, Y.; Wang, D.; Yuan, M. Effects of Fluorinated Aromatic Spacer in Ag–Bi Double Perovskite for X-ray Detector. *J. Phys. Chem. C* **2022**, *126*, 19417-19423.
(5) Connor, B. A.; Leppert, L.; Smith, M. D.; Neaton, J. B.; Karunadasa, H. I. Layered Halide Double Perovskites: Dimensional Reduction of Cs2AgBiBr6. *J. Am. Chem. Soc.* **2018**, *140*, 5235-5240.
(6) Hooijer, R.; Weis, A.; Biewald, A.; Sirtl, M. T.; Malburg, J.; Holfeuer, R.; Thamm, S.; Amin, A. A. Y.; Righetto, M.; Hartschuh, A.; Herz, L. M.; Bein, T. Silver-Bismuth Based 2D Double Perovskites $(4FPEA)_4AgBiX_8$ (X = Cl, Br, I): Highly Oriented Thin Films with Large Domain Sizes and Ultrafast Charge-Carrier Localization. *Adv. Opt. Mater.* **2022**, *10*, 2200354.
(7) McClure, E. T.; Ball, M. R.; Windl, W.; Woodward, P. M. Cs_2AgBiX_6 (X = Br, Cl): New Visible Light Absorbing, Lead-Free Halide Perovskite Semiconductors. *Chem. Mater.* **2016**, *28*, 1348-1354.
(8) Mączka, M.; Zaręba, J. K.; Gągor, A.; Stefańska, D.; Ptak, M.; Roleder, K.; Kajewski, D.; Soszyński, A.; Fedoruk, K.; Sieradzki, A. [Methylhydrazinium]$_2$PbBr$_{-4}$, a Ferroelectric Hybrid Organic–Inorganic Perovskite with Multiple Nonlinear Optical Outputs. *Chem. Mater.* **2021**, *33*, 2331-2342.
(9) Long, L.; Huang, Z.; Xu, Z.-K.; Gan, T.; Qin, Y.; Chen, Z.; Wang, Z.-X. H/F substitution activating tunable dimensions and dielectric–optical properties in organic lead-bromide hybrids. *Inorg. Chem. Front.* **2024**, *11*, 845-852.
(10) Ye, H.-Y.; Liao, W.-Q.; Hu, C.-L.; Zhang, Y.; You, Y.-M.; Mao, J.-G.; Li, P.-F.; Xiong, R.-G. Bandgap Engineering of Lead-Halide Perovskite-Type Ferroelectrics. *Adv. Mater.* **2016**, *28*, 2579-2586.
(11) Xu, L.; Guan, Q.; Ye, H.; Wu, J.; Li, H.; Geng, Y.; Zhang, C.; Zhu, Z.-K.; Han, Z.; Yin, Q.; Luo, J. Bromine-Substituted Cation Anchoring to Suppress Ion Migration in Alternating Cations Intercalation-Type Perovskite for Stable X-Ray Detection. *Small Struct.* **2025**, *6*, 2400449.
(12) Jung, M.-H. White-Light Emission from the Structural Distortion Induced by Control of Halide Composition of Two-Dimensional Perovskites ($(C_6H_5CH_2NH_3)_2PbBr_{4-x}Cl_x$). *Inorg. Chem.* **2019**, *58*, 6748-6757.
(13) Rajput, S. A.; Antharjanam, S.; Chandiran, A. K. Blue emission in a fluorinated 2D $(C_8H_{11}FN)$-$_2PbBr_4$ perovskite with 11.17% photoluminescence quantum yield. *J. Mater. Chem. C* **2025**.
(14) Zhao, X.-H.; Li, N.-N.; Peng, J.; Xu, J.; Luo, P.; Dong, X.-Y.; Hu, X. Circularly polarized luminescence enlargement from crystals to oriented films of enantiopure 2D hybrid perovskites. *Chem. Commun.* **2023**, *59*, 6881-6884.
(15) Li, H.; Zhao, X.-Z.; Yang, H.-R.; Guo, T.-M.; Li, W.; Feng, R. Chiral 2D Halide Perovskites for Piezoelectric Energy Harvesting and Ultrasound Detection. *Eur. J. Inorg. Chem.* **2025**, *28*, e202400612.
(16) Gu, Q.; Chen, K.; Zhang, X.; Wang, S.; Wu, S.; Huang, W. Butylammonium-

Based Chiral 2D Perovskite Single Crystals for Efficient UV Circularly Polarized Light Differentiation and High-Performance X-ray Detection. *ACS Appl. Mater. Interfaces* **2025**, *17*, 17127-17134.
